# A severe asthma phenotype of excessive airway *Haemophilus influenzae* relative abundance associated with sputum neutrophilia

**DOI:** 10.1002/ctm2.70007

**Published:** 2024-08-26

**Authors:** Ali Versi, Adnan Azim, Fransiskus Xaverius Ivan, Mahmoud I Abdel‐Aziz, Stewart Bates, John Riley, Mohib Uddin, Nazanin Zounemat Kermani, Anke‐H Maitland‐Van Der Zee, Sven‐Eric Dahlen, Ratko Djukanovic, Sanjay H Chotirmall, Peter Howarth, Ian M Adcock, Kian Fan Chung, the U‐BIOPRED study group

**Affiliations:** ^1^ National Heart & Lung Institute Imperial College London London UK; ^2^ Respiratory Department, Faculty of Medicine Southampton University Southampton UK; ^3^ Lee Kong Chian School of Medicine Nanyang Technological University Singapore Singapore; ^4^ Department of Pulmonary Medicine Amsterdam University Medical Centers University of Amsterdam Amsterdam Netherlands; ^5^ Respiratory Therapeutic Unit GSK Stockley Park UK; ^6^ AstraZeneca BioPharmaceuticals R&D Gothenburg Sweden; ^7^ Department of Medicine Huddinge Karolinska Institute Stockholm Sweden; ^8^ Department of Respiratory and Critical Care Medicine Tan Tock Seng Hospital Singapore Singapore

**Keywords:** α‐diversity, *Haemophilus influenzae*, metagenome, *Moraxella catarrhalis*, neutrophils, severe asthma, *Tropheryma whipplei*

## Abstract

**Background:**

Severe asthma (SA) encompasses several clinical phenotypes with a heterogeneous airway microbiome. We determined the phenotypes associated with a low α‐diversity microbiome.

**Methods:**

Metagenomic sequencing was performed on sputum samples from SA participants. A threshold of 2 standard deviations below the mean of α‐diversity of mild‐moderate asthma and healthy control subjects was used to define those with an abnormal abundance threshold as relative dominant species (RDS).

**Findings:**

Fifty‐one out of 97 SA samples were classified as RDSs with *Haemophilus influenzae* RDS being most common (*n* = 16), followed by *Actinobacillus unclassified* (*n* = 10), *Veillonella unclassified* (*n* = 9)*, Haemophilus aegyptius* (*n* = 9)*, Streptococcus pseudopneumoniae* (*n = 7*), *Propionibacterium acnes* (*n = 5), Moraxella catarrhalis* (*n *=* 5*) and *Tropheryma whipplei* (*n* = 5). *Haemophilus influenzae* RDS had the highest duration of disease, more exacerbations in previous year and greatest number on daily oral corticosteroids. Hierarchical clustering of RDSs revealed a C2 cluster (*n* = 9) of highest relative abundance of exclusively *Haemophilus influenzae* RDSs with longer duration of disease and higher sputum neutrophil counts associated with enrichment pathways of MAPK, NF‐κB, TNF, mTOR and necroptosis, compared to the only other cluster, C1, which consisted of 7 *Haemophilus influenzae* RDSs out of 42. Sputum transcriptomics of C2 cluster compared to C1 RDSs revealed higher expression of neutrophil extracellular trap pathway (NETosis), IL6‐transignalling signature and neutrophil activation.

**Conclusion:**

We describe a *Haemophilus influenzae* cluster of the highest relative abundance associated with neutrophilic inflammation and NETosis indicating a host response to the bacteria. This phenotype of severe asthma may respond to specific antibiotics.

## Graphical abstract

- The relative dominant species (RDS) in 51 sputum samples of severe asthma patients are described including *Haemophilus influenzae, Moraxella catarrhalis* and *Tropheryma whipplei* RDSs.
- Hierarchical clustering revealed 2 clusters, one entirely composed *of Haemophilus influenzae* with relative abundance of 76.3%.
- This cluster was characterised by long duration of asthma and exacerbations, and enriched NETosis and IL‐6 trans‐signalling pathways.

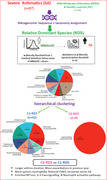

## 1 INTRODUCTION

Molecular phenotypes characterised by either eosinophilic or neutrophilic inflammation have been described from transcriptomic or proteomic analyses of bronchial biopsies or sputum cells from patients with severe asthma.^1^, ^2^ The severe eosinophilic asthma associated with Type 2 inflammatory pathways responds well to biologic therapies targeting the cytokines, interleukin (IL)−4, IL‐5 and IL‐13. However, the phenotypes characterised by a neutrophilic inflammation remain ill‐defined, and such phenotypes may be associated with dysbiosis of the airway microbiome as measured by low diversity, which is described in patients with uncontrolled or severe asthma.^3^, ^4^ Metagenomic analysis of the airway microbiome in asthma has revealed an abundance of *Haemophilus influenzae* and *Moraxella catarrhalis* associated with sputum neutrophilia and linked to inflammasome and neutrophil activation,^5^ in line with studies that have reported neutrophilic inflammation with an enrichment of *Proteobacteria* including *Moraxella* and *Haemophilus*, and in particular *Haemophilus influenzae*.^4^, ^6^, ^7^ Although neutrophils form part of the host mechanism critical for fighting respiratory infections, many opportunistic pathogens have evolved immune mechanisms to evade microbial entrapment and killing.^8^

In order to define further the different phenotypes of severe asthma more closely, we determined whether these could be dominated by any particular bacterial species. In order to do this, we defined the relative dominant bacterial species in each sputum sample collected based on the impairment of the diversity of the bacterial species defined from the metagenomic analysis of the sputum samples in the severe asthma U‐BIOPRED cohort.^9^ Thus, microbial α‐diversity denotes the relative abundance of microbial species in space and time in a biological sample and a decrease has been associated with declining health status.^10^ We examined the host molecular pathways associated with these relative dominant species containing specific bacterial species. In so doing, we have defined a restricted phenotype of severe asthma dominated by the highest abundance of airway *Haemophilus influenzae* with evidence of sputum neutrophilia and host–bacterial interactions.

## 2 METHODS

### 2.1 Participants

The U‐BIOPRED cohort consists of adult severe asthmatics (SA) that included nonsmokers and current and/or ex‐smokers, and two control groups, mild‐moderate asthmatics (MMA) and healthy volunteers (HC), as previously described^9^ (Table S1) at baseline. Twelve months later, a subset of severe asthmatics returned for follow‐up. This study was approved by the Ethics Committee of each participating clinical institution and adhered to the standards set by International Conference on harmonisation and Good Clinical Practice. All patients gave written informed consent to participate in the study.

### 2.2 Sample collection, DNA/RNA extraction and data preprocessing

Induced sputum samples were obtained following inhalation of hypertonic saline and transcriptomic expression was performed using Affymetrix U133 Plus 2.0 (Affymetrix, Santa Clara, CA, USA) microarrays using RNA extracted from sputum cells. Quality checks were performed according to Affymetrix® recommendations and the expression matrix was derived using the robust microarray analysis (RMA) method from the *affy R package*.^11^

Metagenomic sequencing analysis was performed on frozen sputum samples from 147 participants (99 SA, 25 MMA, 23 HC) at baseline and 44 SA participants at follow‐up by Second Genome (San Francisco, California, USA)^5^ (Figure S2). After quality control and host reads removal of the metagenomic data was performed, samples from 97 SA patients, 25 MMA and 23 HC subjects out of the total 145 were used for further analyses. The MetaPhlAn2 pipeline (*version 2.7.2*) and its marker database^12^ was used to estimate microbiome profiles. Metagenomic functional profiling to estimate the abundance of microbial gene families and pathways from metagenomic data was performed using the HUMAnN2^13^ and the UniProt pathway database (UNIPATHWAY database)^14^, ^15^ (Supplementary file, Section 4).

### 2.3 Definition of relative dominant species (RDSs)

**FIGURE 1:**
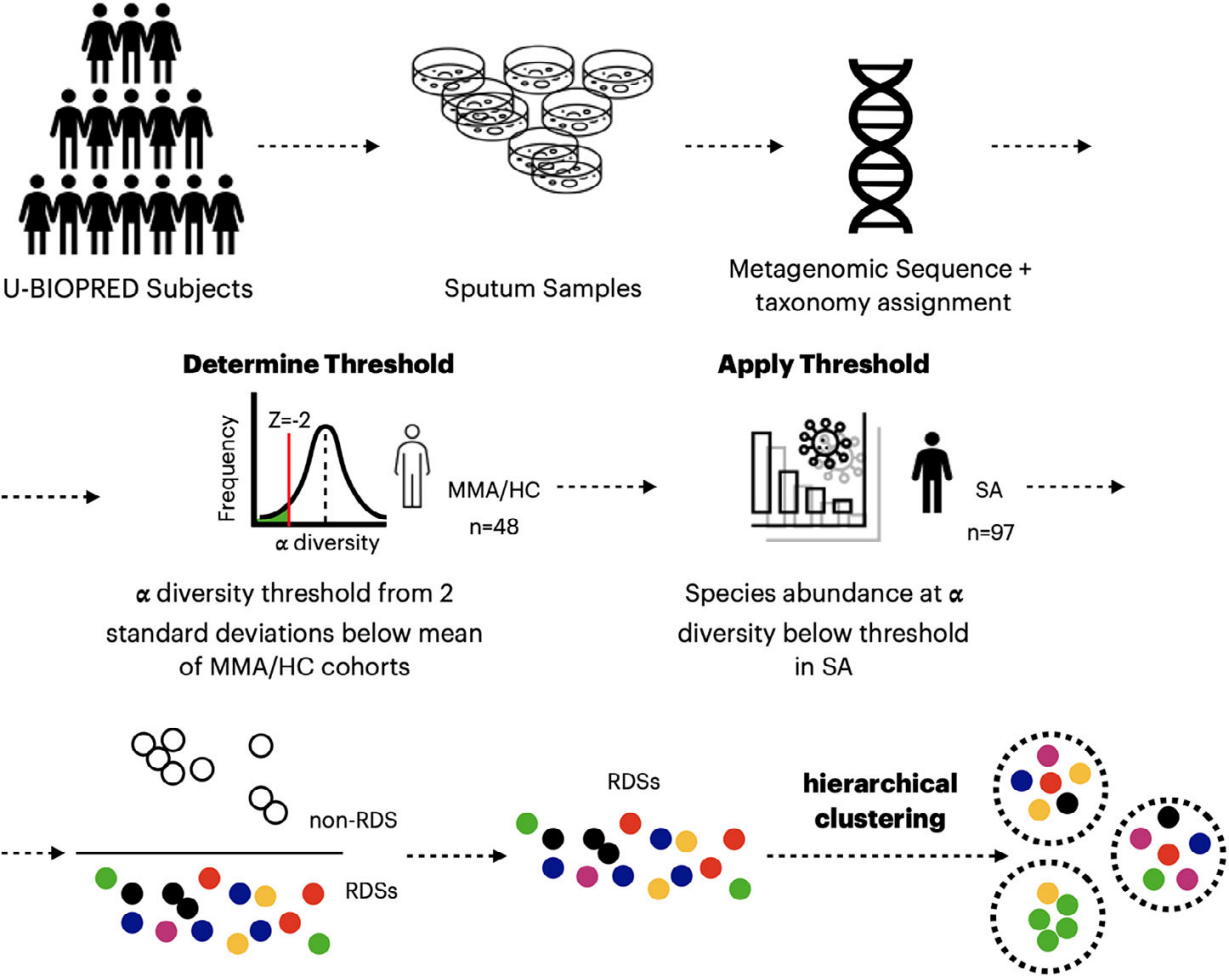
Flow chart showing the determination of relative dominant species (RDS) by α‐diversity threshold in the sputum samples and applied to samples of severe asthma patients and hierarchical clustering of RDS samples. MMA/HC: mild‐moderate asthma/Healthy controls; SA: severe asthma. The numbers studied in each cohort are shown.

The relative abundance of a particular bacterial species was derived from the MetaPhlAn2 pipeline (Supplementary file, Section 3). Using *a* predetermined Shannon's α‐diversity threshold, an abundance cut‐off was determined for each bacterial species based on the minimum abundance of that species associated with the reduction in Shannon's α‐diversity to, or below, this threshold in the SA cohort (Supplementary file, Section 1). For each species, we iterated through a range of abundance values increasing by 0.5 from 0% to 75% and excluded samples that did not have an abundance at or above that value and then calculated the Shannon's α‐diversity of the remaining samples. When the Shannon's α‐diversity of the remaining samples was found to be at or below the predetermined Shannon's α‐diversity threshold, that abundance value was designated as the RDS abundance cut‐off for that species. For samples that contained no RDSs, the species was designated as a non‐RDS. The algorithm is shown in Figure S1. The predetermined Shannon's α‐diversity threshold was calculated using a Z‐Score of −2 of Shannon's α‐diversity obtained from the combined MMA and HC cohorts (Figures 1 and S3).

### 2.4 Determination of dominant RDS

For samples with multiple RDSs, a dominant RDS was defined. A factor was created for each species in each sample by dividing the abundance of each species in a given sample by the specific threshold unique to that species established in the RDS definition. The species in a given sample with the highest factor was considered to be the dominant RDS in that sample.

### 2.5 Clustering on abundance of low‐diversity samples

Clustering by species compositional abundance was performed to understand the heterogeneity of low‐diversity samples. To determine similarity between samples, the Aitcheson distance β‐diversity dissimilarity measure^16^ was employed because it was suited for compositional data, as the variation between sequence reads for each sample is unknown.^17^ This was computed on the species‐level relative abundance from the metagenomics data of the RDS samples in SA. Hierarchical Ward2 agglomerative clustering on the Aitchison distance was performed and the optimum number of clusters determined using the average silhouette width^18^ and the Calinski–Harabasz score.^19^ Consensus clustering^20^ was used to assess stability by resampling, randomly removing 10% of the data and repeating the clustering through 1000 iterations. The empirical cumulative distribution function (CDF) displays the consensus distributions for each value of *k* derived using the ConsensusClusterPlus package.^21^

### 2.6 Longitudinal analysis

Forty‐three out of the 44 follow‐up samples had corresponding baseline samples. This was used to define the stability of dominant RDSs between baseline and follow‐up.

### 2.7 Gene set variation analysis

Gene set variation analysis (GSVA) was performed in R using the Bioconductor GSVA package to estimate sample‐wise enrichment of gene signatures.^22^

### 2.8 Statistical analysis

We used *pandas 1.4.2* ^23^ and *python 3.9.10* ^24^ data analysis tools. Statistical analyses were performed using R version 4.1.1.^25^ Differentially abundant microbiome species between clusters and differentially prevalent microbial pathway were identified using ANCOM‐BC,^26^ which was selected due to its suitability for compositional data.^17^ The Holm–Bonferroni for adjustment was used to control for FDR. Differentially abundant species analysis was performed between clusters generated from the hierarchical clustering of low‐diversity RDS samples and MMA/HC group.

Differentially expressed gene (DEG) analysis was performed on sputum transcriptomics^27^ between different RDS species compared to the MMA and HC groups combined (MMA/HC), and non‐RDSs compared to MMA/HC using limma.^28^ A Benjamini–Hochberg false‐discovery rate (FDR) adjustment was applied with FDR < 0.05 and absolute log_2_ fold‐change ≥0.5 were considered statistically significant in transcriptomic analyses. DEG results were used to perform pathway enrichment using ClusterProfiler.^29^ Comparison of the clinical variables between groups was performed using analysis of variance (ANOVA) for multiple group comparison of normally distributed variables. Kruskal–Wallis test was used for multiple group comparison of nonnormally distributed variables or ordered categorical and chi‐squared test was used for qualitative variables.

## 3 RESULTS

Figure S2 shows the Consort diagram with a final repartition of the 97 severe asthma subjects into 2 clusters of RDSs.

### 3.1 Low α‐diversity RDSs

The distribution and threshold of the relative dominant species in RDSs are shown in Figure S13. Patients with low α‐diversity RDSs had higher sputum neutrophils compared to those with normal diversity with lower sputum macrophages and a greater prevalence of allergic rhinitis (Table S2). Twenty‐four species were categorised as being RDSs. The most common RDSs in descending order were *Haemophilus influenzae* (16 samples), *Actinobacillus unclassified* (*n* = 10), *Veillonella unclassified* (*n* = 9)*, Haemophilus aegyptius* (*n* = 9)*, Streptococcus pseudopneumoniae* (*n *= 7), *Propionibacterium acnes* (*n *= 5), *Moraxella catarrhalis* (*n *= 5) and *Tropheryma whipplei* (*n* = 5) (Table 1). Fifty‐one out of 97 samples contained one or more RDSs but the RDSs were generally mutually exclusive, with 29 samples containing only one RDS, 10 samples two RDSs, 9 samples three RDSs and 3 samples four RDSs (Figures S4A and S5). *Haemophilus aegyptius* RDSs always co‐occurred with *Haemophilus influenzae* RDSs or the *Actinobacillus unclassified* RDS. The *Actinobacillus unclassified* RDS generally co‐occurred with the *Haemophilus influenzae* RDS *(7 samples). Pseudomonas aeruginosa and Pseudomonas unclassified RDSs* were seen in 1 sample and co‐occurred with the *Haemophilus aegyptius* RDS and *Haemophilus influenzae* RDS*. Moraxella catarrhalis* RDS only co‐occurred with one or more RDS species in 1 sample, which were the RDS species *Haemophilus aegyptius* and *Haemophilus influenzae. Tropheryma whipplei* RDS co‐occurred with the *Streptococcus pseudopneumoniae* RDS in 1 sample.

**TABLE 1:** Presence and relative dominance of bacterial species in sputum of severe asthma.

Species	Phylum	RDS samples (*n* = 51)	Presence (% of samples with detectable species)	Mean abundance (%)^*^	Mean abundance in RDS samples (%)^*^
*Haemophilus influenzae*	Proteobacteria	16	44.33 (43/97)	18.40 ± 31.73	48.23 ± 36.14
*Actinobacillus unclassified*	Proteobacteria	10	47.42 (46/97)	2.00 ± 2.73	5.62 ± 4.05
*Veillonella unclassified*	Firmicutes	9	87.63 (85/97)	14.00 ± 17.10	50.53 ± 30.96
*Haemophilus aegyptius*	Proteobacteria	9	17.53 (17/97)	2.02 ± 2.07	3.40 ± 1.98
*Streptococcus pseudopneumoniae*	Firmicutes	7	41.24 (40/97)	8.48 ± 17.04	40.05 ± 21.40
*Moraxella catarrhalis*	Proteobacteria	5	10.31 (10/97)	24.98 ± 37.50	49.09 ± 41.35
*Tropheryma whipplei*	Actinobacteria	5	14.43 (14/97)	22.13 ± 25.67	53.71 ± 12.31
*Propionibacterium acnes*	Actinobacteria	5	14.43 (14/97)	7.24 ± 16.22	19.77 ± 23.45
*Streptococcus mitis oralis pneumoniae*	Firmicutes	4	69.07 (67/97)	3.80 ± 6.82	26.10 ± 5.71
*Neisseria unclassified*	Proteobacteria	2	56.70 (55/97)	5.46 ± 8.22	38.98 ± 4.06
*Rothia dentocariosa*	Actinobacteria	2	53.61 (52/97)	2.11 ± 5.65	28.79 ± 6.08
*Pseudomonas aeruginosa*	Proteobacteria	1	1.03 (1/97)	23.83	23.83
*Porphyromonas gingivalis*	Bacteroidetes	1	20.62 (20/97)	13.04 ± 18.85	75.01
*Haemophilus parainfluenzae*	Proteobacteria	1	75.26 (73/97)	6.18 ± 6.80	46.75
*Porphyromonas endodontalis*	Bacteroidetes	1	38.14 (37/97)	5.48 ± 12.25	66.76
*Pseudomonas unclassified*	Proteobacteria	1	1.03 (1/97)	4.23	4.23
*Alloprevotella unclassified*	Bacteroidetes	1	23.71 (23/97)	3.02 ± 6.71	32.32
*Deinococcus unclassified*	Deinococcus Thermus	1	11.34 (11/97)	1.72 ± 5.26	17.57
*Kingella unclassified*	Proteobacteria	1	34.02 (33/97)	1.63 ± 5.32	30.86
*Prevotella baroniae*	Bacteroidetes	1	15.46 (15/97)	1.53 ± 3.49	12.70
*Fusobacterium nucleatum*	Fusobacteria	1	35.05 (34/97)	1.03 ± 2.15	12.16
*Comamonas unclassified*	Proteobacteria	1	3.09 (3/97)	0.53 ± 0.90	1.57
*Prevotella dentalis*	Bacteroidetes	1	14.43 (14/97)	0.50 ± 0.80	3.11
*Eimeria tenella*	Apicomplexa	1	5.15 (5/97)	0.26 ± 0.50	1.15

*Note*: Presence denotes the percentage of samples where a particular species exists with a greater than zero abundance. Abundance is the median relative abundance of all the samples for a particular species where it exists with a greater than zero abundance.

* Data shown as mean ± SD.

Abbreviation: RDS: relative dominant species.

### 3.2 *Haemophilus influenzae*, *Moraxella catarrhalis* and *Tropheryma whipplei* RDSs

#### 3.2.1 *Haemophilus influenzae* RDS

*Haemophilus influenzae* was detectable in 44% (43/97) of SA samples with a mean relative abundance of 1.42% ± 13.5%. 37% (16/43) of samples with *Haemophilus influenzae* met the loss of diversity criteria, becoming an RDS. The mean relative abundance of *Haemophilus influenzae* within RDS samples was 48.23 ± 36.14. In 51% of samples with low diversity, *Haemophilus influenzae* was not identified. Patients with *Haemophilus influenzae* RDS had the longest duration of disease with evidence of more severe disease with highest number of exacerbations in the previous year and of patients on oral corticosteroid (OCS) therapy amongst these 3 RDSs (Table 2). They had the second highest sputum neutrophil count after the *Moraxella catarrhalis* RDSs (*p* < .05 compared with non‐RDSs).

**TABLE 2:** Characteristics of severe asthma with *Haemophilus influenzae* (*Hi*), *Moraxella catarrhalis* (*Mc*) and *Tropheryma whipplei* (*Tw*) relative dominant species (RDS).

	*Hi* RDS	*Mc* RDS	*Tw* RDS	Non‐RDSs	MMA	*p* Value	^#^ *p* Value	^##^ *p* Value
Subjects, *n*	15	4	5	46	25	NA	NA	NA
Age (years)	51.0 [45.5, 56.5]	57.5 [48.2, 62.0]	62.0 [46.0, 71.0]	55.0 [45.2, 62.8]	45.0 [28.0, 51.0]	<.005	NS	NS
Females, *n* (%)	9 (60.0)	3 (75.0)	3 (60.0)	29 (63.0)	12 (48.0)	NS	NS	NS
BMI	26.4 (2.6)	27.7 (4.5)	29.9 (5.0)	28.8 (6.3)	25.6 (4.8)	NS	NS	NS
Duration years,	38.2 (14.0)	18.0 (21.5)	25.0 (13.0)	22.4 (16.1)	23.5 (16.6)	<.05	<.05	<.05
Current smoking, *n* (%)	2 (13.3)	0 (0)	0 (0)	6 (13.0)	0 (0)	<.05	NS	NS
Oral corticosteroid daily, *n* (%)	7 (46.7)	1 (25.0)	1 (20.0)	17 (37.0)	0 (0)	<.01	NS	Ns
ACQ‐5 score,	2.2 (1.3)	2.9 (1.6)	2.8 (1.1)	2.3 (1.2)	1.1 (0.8)	<.001	NS	NS
Exacerbations, per year,	2.9 (2.5)	1.0 (0.8)	1.2 (1.3)	2.4 (2.1)	0.6 (1.0)	<.001	NS	NS
Nasal polyposis, *n* (%)	4 (26.7)	2 (50.0)	2 (40.0)	15 (32.6)	4 (16.0)	NS	NS	NS
Eczema, *n* (%)	6 (40.0)	1 (25.0)	0 (0)	11 (23.9)	9 (36.0)	NS	NS	NS
Allergic rhinitis, *n* (%)	8 (53.3)	3 (75.0)	1 (20.0)	11 (23.9)	13 (52.0)	NS	NS	NS
GERD, *n* (%)	6 (40.0)	1 (25.0)	2 (40.0)	18 (39.1)	3 (12.0)	NS	NS	NS
Blood eosinophils µL^−1^	0.3 (0.2)	0.5 (0.6)	0.4 (0.2)	0.3 (0.3)	0.2 (0.1)	NS	NS	NS
Blood neutrophils µL^−1^	6.5 (2.5)	6.8 (4.4)	4.8 (1.5)	5.1 (2.6)	3.7 (1.2)	<.005	NS	NS
Sputum neutrophils (%),	68.0 (27.7)	82.1 (25.4)	56.2 (11.0)	49.5 (24.0)	48.0 (24.5)	<.05	<.05	NS
Sputum eosinophils (%)	19.4 (25.2)	1.4 (1.8)	19.7 (13.2)	13.1 (20.5)	3.8 (11.1)	NS	NS	NS
Sputum lymphocytes (%)	0.8 (0.6)	0.7 (0.5)	1.2 (1.3)	1.6 (1.4)	2.3 (2.5)	NS	NS	NS
Sputum macrophage (%)	11.8 (8.3)	15.8 (23.2)	22.8 (18.8)	35.7 (22.5)	46.5 (23.9)	<.001	.001	NS
FEV1 (% predicted)	65.1 (26.9)	62.2 (21.1)	40.3 (10.2)	67.4 (18.7)	90.5 (19.0)	<.001	NS	NS
FeNO (ppb)	30.8 (28.7)	22.2 (10.9)	28.2 (20.1)	40.1 (38.6)	39.6 (32.7)	NS	NS	NS
Smoking pack years	16.8 (11.4)	0 (0)	5.7 (3.3)	18.3 (18.4)	3.4 (1.5)	NS	NS	NS
Antibiotic current, *n* (%)	2 (13.3)	0 (0)	1 (20.0)	10 (21.7)	0 (0)	NS	NS	NS
CRP (mg/L)	10.0 (16.6)	3.6 (1.5)	2.7 (1.8)	6.6 (15.3)	1.6 (2.3)	NS	NS	NS
IL‐1α (pg/mL)	34.5 (10.7)	34.8 (6.2)	36.7 (8.3)	35.1 (6.4)	31.9 (6.6)	NS	NS	NS
IL‐6 (pg/mL)	1.3 (1.3)	1.6 (0.6)	1.1 (0.4)	1.1 (0.8)	0.7 (0.7)	NS	NS	NS
Serum IL‐8 (pg/mL)	7.6 (12.7)	3.0 (1.4)	3.1 (0.6)	4.0 (3.0)	3.5 (1.4)	NS	NS	NS
Serum C5a (pg/mL)	60.9 (27.3)	36.7 (9.9)	51.5 (76.0)	39.8 (19.6)	34.4 (18.5)	NS	NS	NS

*Note*: Only samples with no co‐occurring RDSs of the three species (*Hi*, *Mc*, *Tw*) were selected. Data shown as mean (standard deviation) unless variable is categorical where *n* (%). Age shown as median [95% confidence intervals]. *p* Value: comparing RDSs and non‐RDSs.

*p* Value: comparing the five groups: *Hi* RDS, *Mc* RDS, *Tw* RDS, non‐RDSs, MMA.

^#^ *p* Value: comparing the four groups: *Hi* RDS, *Mc* RDS, *Tw* RDS, non‐RDSs.

^##^ *p* Value: comparing the three cluster groups: *Hi* RDS, *Mc* RDS, *Tw* RDS.

Abbreviations: ACQ5: Asthma Control Questionnaire score; BMI: body mass index; CRP: serum C‐reactive protein; FeNO: fractional exhaled nitric oxide; FEV1: forced expiratory volume in the first second pre‐salbutamol; FVC: forced vital capacity; GERD: gastroesophageal reflux disease; MMA: mild‐moderate asthma; N/A: not applicable; NS: not significant; ppb: parts per billion.

**FIGURE 2:**
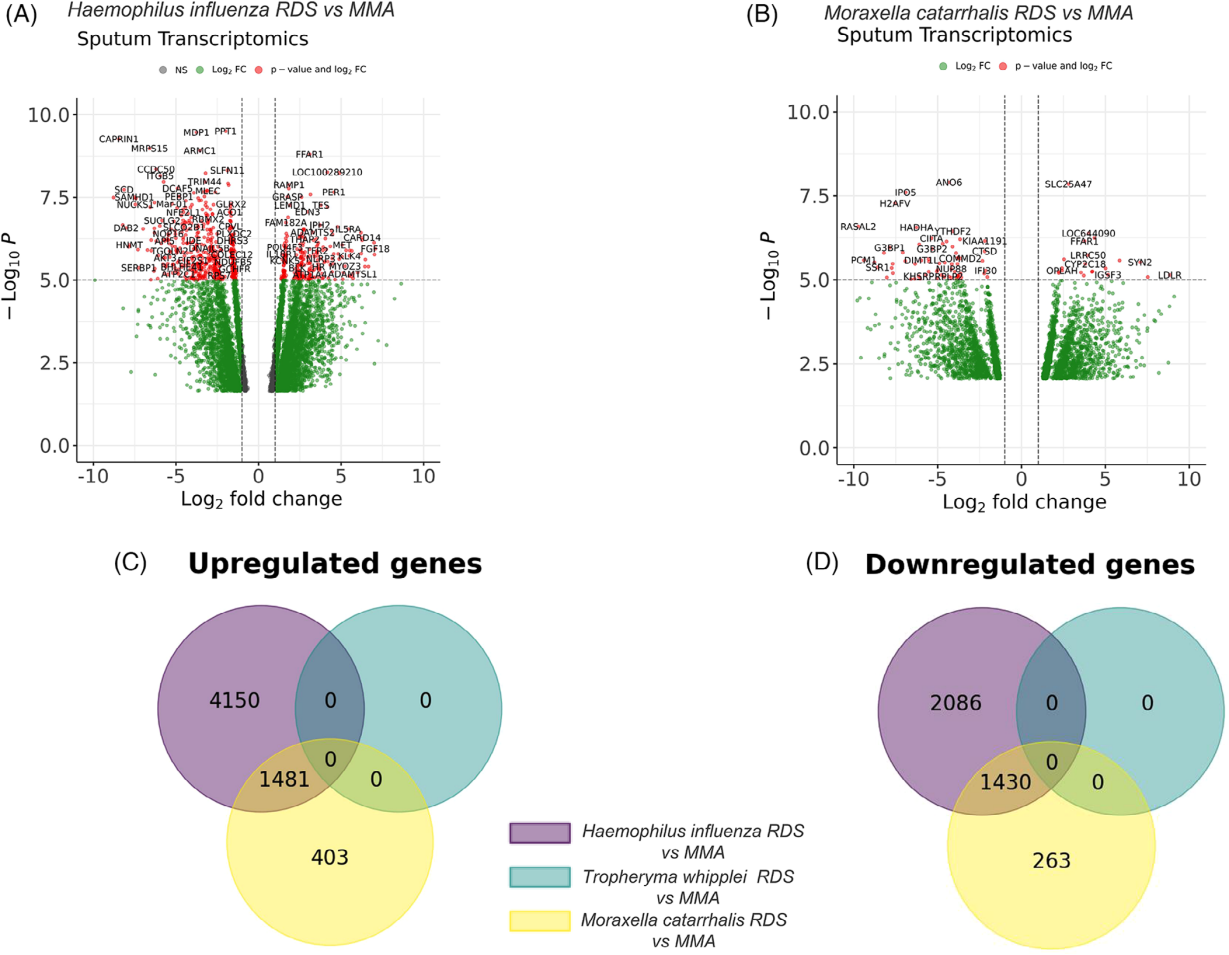
**Transcriptomic differentially expressed genes between RDS groups and MMA/HC cohort**. Volcano plot depicting the sputum transcriptomic DEG between (**A)** *Haemophilus influenzae* RDS vs. MMA, (**B**) *Moraxella catarrhalis* RDS vs. MMA, Venn diagram depicts the overlap between upregulated genes (**C**) and downregulated genes (D) from the differentially expressed genes of the sputum transcriptomics between the *Haemophilus influenzae*, *Moraxella catarrhalis* and *Tropheryma whipplei* RDSs and MMA. MMA: mild‐moderate asthma.

Relative to the patients with RDSs, sputum transcriptomics identified few DEGs differentially expressed between severe asthma patients with normal diversity and MMA/HC groups. There were no DEGs in sputum transcriptomics between patients with RDSs compared to non‐RDSs. Patients with *Haemophilus influenzae* RDS have 5631 more genes upregulated compared to MMA and non‐*Hi* RDSs (Figure 2A). Pathway analysis of DEGs showed enrichment of positive regulation of natural killer cell‐mediated cytotoxicity, positive regulation of type 2a hypersensitivity, antigen processing and toll‐like receptor signalling pathway compared to the MMA group (Figure S6A).

#### 3.2.2 *Moraxella catarrhalis* RDS

*Moraxella catarrhalis* was detectable in 10% (10/97) of severe asthmatic samples with a mean abundance of 24.98 ± 37.5% and 50% (5/10) of samples met the loss of diversity criteria of RDS. The mean abundance of *Moraxella catarrhalis* within RDS samples was 49.09 ± 41.35%. Patients with *Moraxella catarrhalis* RDS had the shortest duration of disease but had the highest sputum neutrophils (Table 2). Sputum eosinophils were low at 1.4% (*p* = .001 compared with non‐RDSs comparator) and FeNO was also reduced (*p* < .05). In addition, these subjects had less exacerbations per year (*p* < .05 compared with non‐RDSs). Patients with *Moraxella catarrhalis* RDS had 1884 genes upregulated compared to MMA (Figure 2B and C). Gene ontology analysis of DEGs showed positive regulation of granulocyte colony‐stimulating factor and macrophage colony stimulating factor production and negative regulation of LPS‐mediated signalling pathway and IL‐12 and IL‐13 production compared to MMA (Figure S6B)

#### 3.2.3 *Tropheryma whipplei* RDS

*Tropheryma whipplei* was detected in 14% (14/97) of severe asthmatic samples with an abundance of 22.13 ± 25.67%. 36% (5/14) of samples with *Tropheryma whipplei* were RDSs. The mean abundance of *Tropheryma whipplei* within RDS samples was 53.71 ± 12.31%. Sputum neutrophils and eosinophils were not different in *Tropheryma whipplei* RDS patients compared to non‐RDS subjects (Table S3). FEV1 was reduced in *Tropheryma whipplei* RDS (*p* = .001 compared with non‐RDSs). Patients with *Tropheryma whipplei* RDS had no upregulated or downregulated genes compared with MMA (Figure 2C–E).

#### 3.2.4 Other species RDSs

Characteristics of patients with sole RDSs of *Actinobacillus unclassified, Streptococcus pseudopneumoniae* and *Veillonella unclassified* are shown in Table S4. While there was no difference between *Veillonella* and non‐RDSs, there were more obese mainly nonsmoking severe asthmatics with higher serum C5a in the *Actinobacillus* RDSs. However, the numbers in these RDSs were small.

### 3.3 Cluster analysis of low diversity samples

**FIGURE 3:**
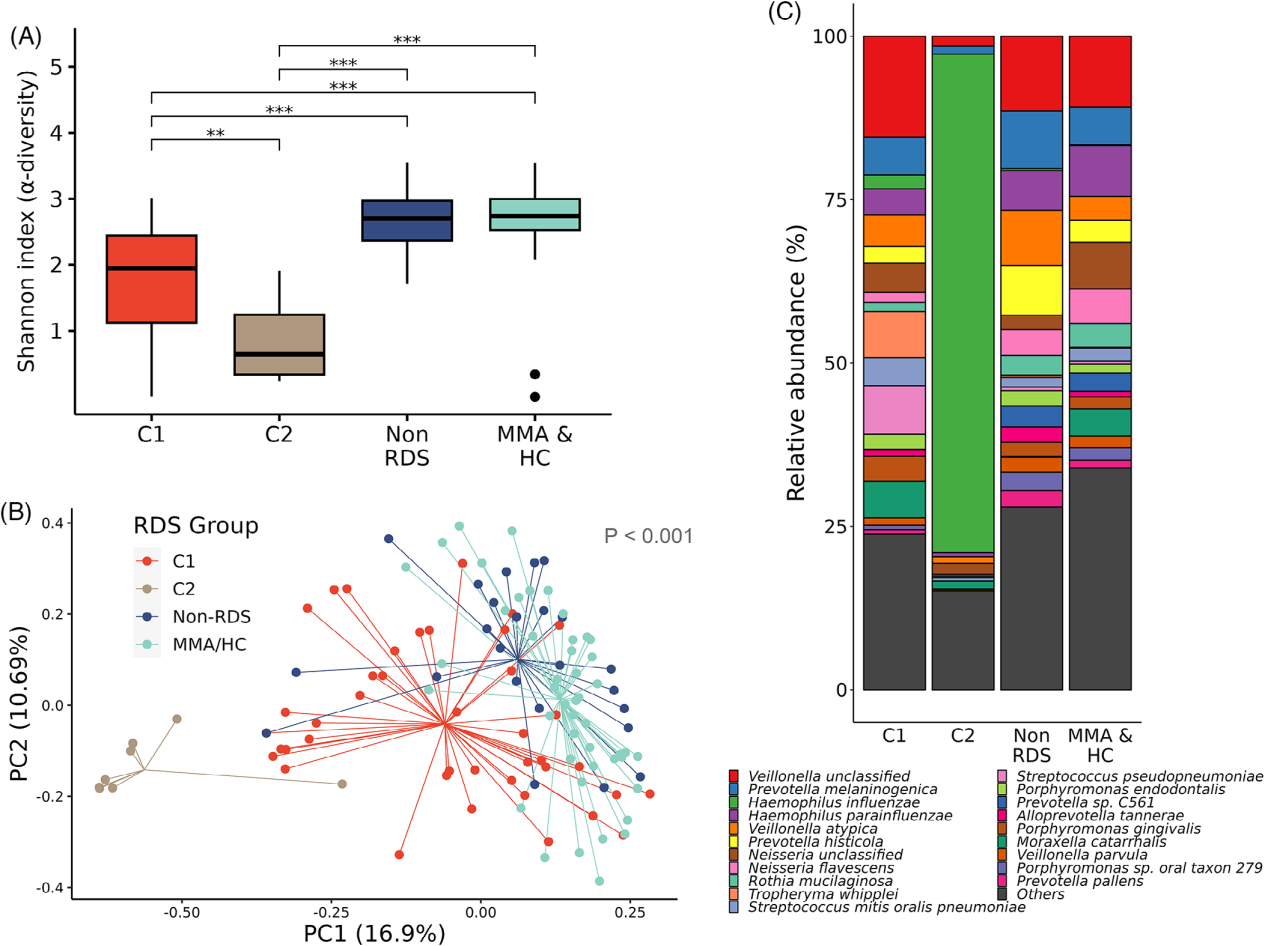
**Diversity of Aitchison distance clusters of RDS samples**. α‐diversity using Shannon's index of each of the clusters C1 and C2 with non‐RDS and MMA/HC groups (**A**). β‐diversity of the clusters with non‐RDS and MMA/HC groups (**B)**. Species‐level relative abundance of each of the 2 clusters compared to that of non‐RDS and MMA/HC groups (**C**).

Cluster analysis of samples with low diversity identified two clusters as demonstrated on silhouette width (C1 and C2) (Figure S7A and B) and by Calinski–Harabasz score (Figure S7C). Consensus clustering also identified stability with 2 clusters (Figure S7D and E) with C2 having the lowest Shannon α‐diversity (Figure 3A), with the greatest degree of separation shown on the principal component analysis plot (Figure 3B). The bee‐swarm plot on Figure S11 shows no difference in composition between C1 and C2 clusters and non‐RDS in terms of nonhuman sequence reads.

**FIGURE 4:**
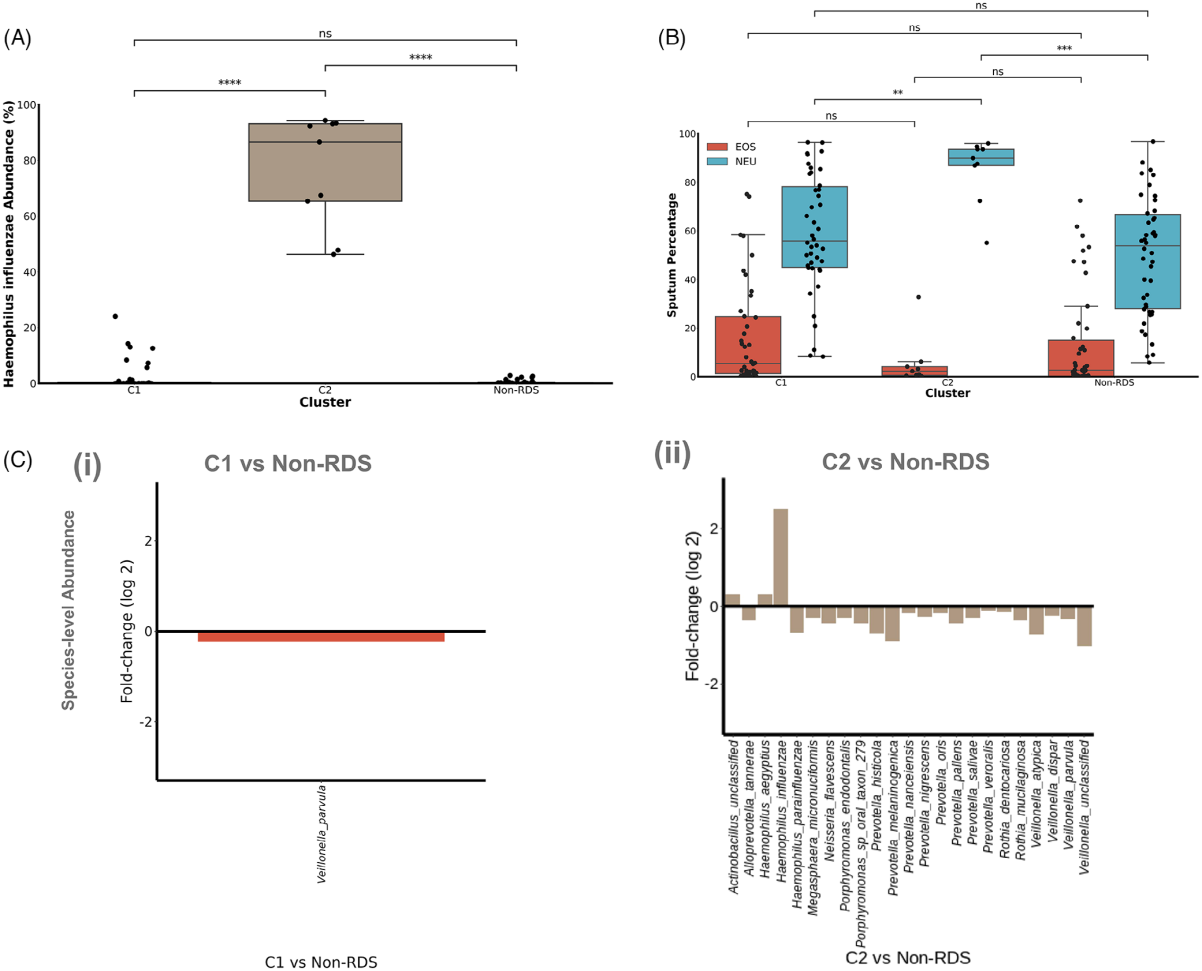
***Haemophilus influenzae* component of C1 and C2 clusters of RDS samples. (A)** *Haemophilus influenzae* abundance of C1 and C2 with non‐RDS. (**B)** Sputum eosinophils and sputum neutrophils (%) of C1 and C2 with non‐RDS. (**C)** Composition log fold‐change between each of the C1 (i) and C2 (ii) clusters versus non‐RDS group. RDS: relative dominant species of severe asthma groups; MMA/HC: mild‐moderate asthma and healthy controls.

C1 had the highest α‐diversity with greatest similarity to MMA and HC subjects combined (MMA/HC), corroborated by the differential abundance analysis with minimal changes in species abundance (Figure 3A and B). C2 was characterised by a large increased abundance of *Haemophilus influenzae* of 76.3 ± 19.9% compared to 2.1 ± 5.1% for C2 (Figure 4A; Table S5). By contrast, *Tropheryma whipplei* and *Moraxella catarrhalis* were more abundant in C1 than in C2.

*Haemophilus influenzae‐*exclusive C2 cluster had a longer disease duration compared to C1 (*p* ≤ .005) (Table 3). Sputum neutrophil counts (%) were higher in C2 (*p* ≤ .001) but sputum eosinophil counts (%) were higher in C1 (*p* ≤ .05) and sputum macrophages (%) lower in C2 (*p* ≤ .001) (Figure 4B). Serum IL‐8 was higher in C2 compared to C1 and non‐RDS groups (*p* < .05) (Table 3). Gene ontology pathway analysis of DEGs of sputum between C1 and C2 clusters showed enrichment of pathways in C2 over C1 related to activated MAPK, NF‐κB, TNF and mTOR signalling pathways and to necroptosis (Figure S8).

**TABLE 3:** Characteristics of clusters C1 and C2 and non‐RDSs.

	C1	C2	Non‐RDSs	^#^ *p* Value	^##^ *p* Value
Subjects, *n*	42	9	46	ND	ND
Age (years)	54.5 [47.0, 59.8]	53.0 [46.0, 55.0]	55.0 [45.2, 62.8]	NS	NS
Females, *n* (%)	21 (50.0)	6 (66.7)	29 (63.0)	NS	NS
BMI	28.7 (5.2)	28.0 (4.5)	28.8 (6.3)	NS	NS
Duration (years)	24.9 (15.3)	41.6 (12.4)	22.4 (16.1)	<.005	<.005
Current smoking, *n* (%)	4 (9.5)	1 (11.1)	6 (13.0)	NS	NS
Oral corticosteroid daily, *n* (%)	18 (42.9)	4 (44.4)	17 (37.0)	NS	NS
ACQ‐5 score	2.3 (1.3)	2.3 (1.1)	2.3 (1.2)	NS	NS
Exacerbations, per year	1.8 (2.1)	3.4 (2.2)	2.4 (2.1)	<.05	NS
Nasal polyposis, *n* (%)	16 (38.1)	2 (22.2)	15 (32.6)	NS	NS
Eczema, *n* (%)	9 (21.4)	5 (55.6)	11 (23.9)	NS	NS
Allergic rhinitis, *n* (%)	20 (47.6)	5 (55.6)	11 (23.9)	<.05	NS
GERD, *n* (%)	19 (45.2)	5 (55.6)	18 (39.1)	NS	NS
Blood eosinophils µL^−1^	0.3 (0.3)	0.3 (0.2)	0.3 (0.3)	NS	NS
Blood neutrophils µL^−1^	5.5 (2.4)	6.8 (2.7)	5.1 (2.6)	NS	NS
Sputum neutrophils (%)	58.9 (24.2)	85.5 (13.5)	49.5 (24.0)	<.001	<.001
Sputum eosinophils (%)	16.4 (21.4)	5.6 (10.4)	13.1 (20.5)	NS	<.05
Sputum lymphocytes (%)	1.1 (1.1)	0.6 (0.4)	1.6 (1.4)	<.05	<.05
Sputum macrophage (%)	23.6 (19.1)	8.2 (7.9)	35.7 (22.5)	<.001	.001
FEV1 (% predicted)	63.9 (20.1)	55.6 (26.5)	67.4 (18.7)	NS	NS
FeNO (ppb)	38.3 (37.6)	19.8 (8.7)	40.1 (38.6)	NS	<.01
Smoking pack years	15.2 (16.7)	15.9 (12.9)	18.3 (18.4)	NS	NS
Antibiotic current, *n* (%)	5 (11.9)	2 (22.2)	10 (21.7)	NS	NS
Serum CRP (mg/L)	6.1 (12.5)	9.1 (10.5)	6.6 (15.3)	NS	NS
Serum IL‐1α (pg/mL)	36.7 (6.3)	31.3 (12.6)	35.1 (6.4)	NS	NS
Serum IL‐6 (pg/mL)	1.4 (1.1)	1.5 (1.5)	1.1 (0.8)	NS	NS
Serum IL‐8 (pg/mL)	3.7 (1.4)	9.6 (15.8)	4.0 (3.0)	<0.05	NS
Serum C5a (pg/mL)	49.0 (30.5)	56.7 (18.9)	39.8 (19.6)	NS	NS

*Note*: Data shown as mean (standard deviation) unless variable is categorical where *n* (%). Age shown as median [95% confidence intervals]. *p* value: comparing RDSs and non‐RDSs.

^#^ *p* value: Chi‐squared tests *p*‐value comparing the three groups: C1‐RDS, C2‐RDS, non‐RDSs.

^##^ *p* value: Chi‐squared tests *p*‐value comparing the two cluster groups: C1‐RDS, C2‐RDS.

Abbreviations: ACQ5: Asthma Control Questionnaire score; BMI: body mass index; CRP: serum C‐reactive protein; FeNO: fractional exhaled nitric oxide; FEV1: forced expiratory volume in the first second pre‐salbutamol; FVC: forced vital capacity; GERD: gastroesophageal reflux disease; N/A: not applicable; NS: not significant; ppb: parts per billion.

Using gene set variation analysis (GSVA), there was a higher expression score of the gene signature of NETosis,^30^ IL6‐transignalling signature^31^ and neutrophil activation while there was lower expression for the oxidative phosphorylation (OXPHOS) and lung tissue resident macrophage in the sputum transcriptome of C2 compared to C1 (Figure S9). There were no significant differences for the gene signatures representing Th17 activation,^32^ blood eosinophil activation and innate lymphoid cell type‐2.

**FIGURE 5:**
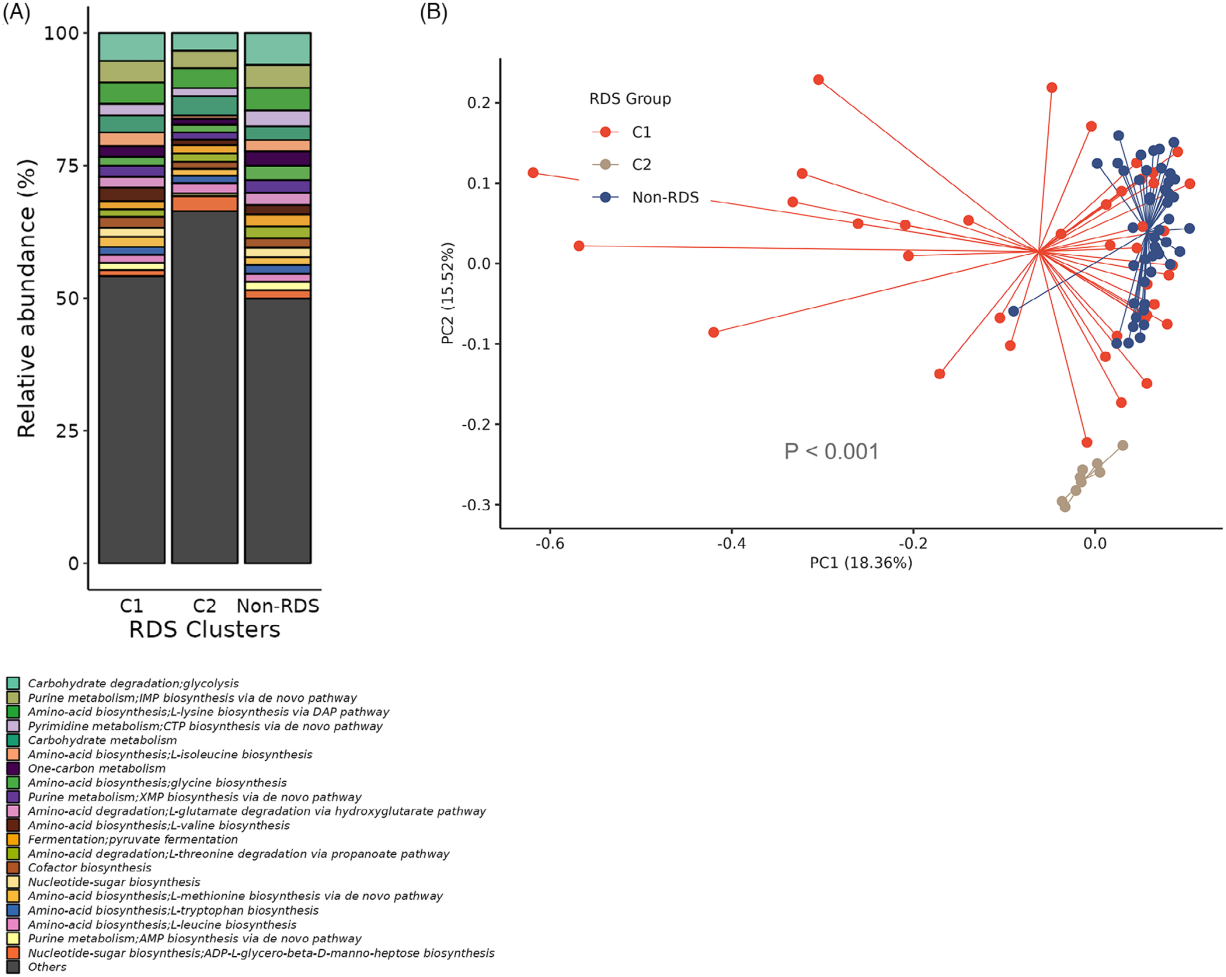
**Microbial pathway prevalence in C1 and C2 cluster samples**. (A) Microbial pathway relative abundance of C1 and C2 clusters compared to that of non‐RDS. (B) β‐diversity of the relative abundant microbial pathways of the C1 and C2 clusters with non‐RDS.

Metagenomic functional analysis demonstrates a distinct microbial pathway profile between C1 and C2 (Figure 5A and B) with PERMANOVA of the Bray‐Curtis dissimilarity matrix being different (*p* < .001). Differentially abundant pathway analysis using ANCOM‐BC showed that C2 had 119 significantly differentially abundant UniProt subpathways compared to C1 (Figure S10), with differences in amino acid biosynthesis and carbohydrate metabolism and degradation.

### 3.4 Longitudinal analysis of severe asthma samples

From the follow‐up cohort, patients with low α‐diversity had lower blood eosinophils compared to those with normal diversity (Table S2). 24 samples were categorised as being low diversity RDS samples and 19 as non‐RDS samples, with 19 samples containing one RDS and 5 samples with two RDSs (Figure S2B). Figure S11 depicts the stability of the dominant RDSs between the baseline and follow‐up time‐point.

At one‐year follow‐up, 7 of the 22 non‐RDS samples became RDS with dominance of *Pseudomonas aeruginosa*, *Haemophilus aegyptius*, *Haemophilus parainfluenzae*, *Streptococcus mitis oralis pneumoniae* and *Veillonella* unclassified. Thirteen of 21 RDSs samples changed RDS status in terms of bacterial species with a shift for *Moraxella catarrhalis* (2 out of 2), *Haemophilus influenzae* (2 out of 5) and for *Tropheryma whipplei* (1 out of 3).

## 4 DISCUSSION

We have quantified the relative abundance of bacterial species in sputum of severe asthma patients by linking it to a state of significantly low diversity defined according to the diversity variance of the mild‐moderate asthma and healthy subjects. This approach was taken because lowly abundant taxa are highly vulnerable to major shifts in abundance^33^, ^34^ and often disproportionately contribute to the structure and function of their community.^35^, ^36^ We have therefore determined the sputum samples of those with severe asthma that had a high relative abundance of a particular species labelled as a RDS, in order to focus on the species that are prone to these major abundance shifts. Low α‐diversity was accompanied by outgrowths of 25 bacterial species for which many are of unknown pathogenic significance. Of these species known to have pathogenic effects in airways diseases such as asthma and COPD, *Haemophilus influenzae* was the most prevalent to constitute a RDS (44%), followed by *Moraxella catarrhalis* and *Tropheryma whipplei* (14.4% each). *Haemophilus influenzae* RDS was associated with longest duration of asthma, highest number of exacerbations and highest use of daily OCS therapy, together with high sputum neutrophilia and lower sputum macrophages, with the presence of sputum eosinophilia only in some subjects. On the other hand, the *Moraxella catarrhalis* RDS was solely associated with sputum neutrophilia but low sputum eosinophils and FeNO and the highest ACQ‐5 score whilst the *Tropheryma whipplei* RDS was characterised by the presence of type 2 inflammation and severe airflow obstruction.

Furthermore, clustering of the bacterial RDSs identified a small but select cluster of the highest relative abundance of *Haemophilus influenzae* who had a long duration of asthma, with sputum neutrophilia, and eosinophil counts and low FeNO levels but with higher levels of serum IL‐8, supporting further the association of increased abundance of *Haemophilus influenzae* with a neutrophilic inflammatory response and low T2 inflammation. Notably, the C2 cluster was also associated with a deficiency of sputum macrophages potentially limiting the efficient clearance of *Haemophilus influenzae* and this might explain the increased bacterial colonisation of the lungs observed. Thus, the C2 cluster membership of exclusively *Haemophilus influenzae* RDSs represented a neutrophilic phenotype with lesser eosinophilic inflammation than the *Haemophilus influenzae* RDSs of the C1 cluster. More importantly, for the first time, we have linked this C2 *Haemophilus influenzae* RDSs cluster with enrichment of activated pathways associated with MAPK, NF‐*k*B, TNF and mTOR, and of cellular necroptosis, which are consistent with previously reported intracellular activation pathways caused by *Haemophilus influenzae* on airway epithelial cells.^37^, ^38^ We propose that the sputum neutrophilia results as a reflection of the host response to detection of the very high relative abundance of *Haemophilus influenzae* as supported by the presence NETosis which has been recognised as a function of activated neutrophils.^39^ It is also possible that the presence of neutrophilic inflammation might create a more favourable environment for the expansion of the *Haemophilus influenzae* species.

More importantly, the gene signature for neutrophil extracellular traps (NETs) was highly expressed in this cluster C2 compared to the C1 cluster using pathway analysis of differentially expressed genes and gene set variation analysis, indicating that a host–bacterial response to the presence of high relative abundance (> 45%) of *Haemophilus influenzae* in the airways. NETs are web‐like scaffolds of DNA complexing with histones and neutrophil granular proteins released from neutrophils upon activation by proinflammatory stimuli, including IL‐8 and TNF, and downstream kinases such as MAPK and mTOR. Excessive NETs in severe asthma may perpetuate NETopathic inflammation by damaging the airway epithelium.^40^ NETs in the context of *Haemophilus influenzae* infection in COPD can also amplify inflammation through the IL6‐transignalling pathway, which was increased in the C2 cluster.^41^ Similarly, with IL‐8 levels found to be elevated in the serum of C2 cluster individuals, this potent chemokine can also induce NET formation in COPD neutrophils via CXCR2 receptor activation.^42^

On the microbial pathways that differentiated the C2 versus the C1 clusters, we found pathways related to carbohydrate biosynthesis and metabolism, some of which were related to glycolysis. This raises the possibility that *Haemophilus influenzae* might be using the glycolytic pathway as energy source for growth, as they were the most relatively abundant bacterial species (mean of 76%) and as it is known that *Haemophilus influenzae* are able to catabolise glucose during both aerobic and anaerobic growth.^43^ The supply of glucose in airway surface liquid glucose may be increased in chronic respiratory diseases such as asthma,^44^ and in the C2 cluster, 2 of the 9 subjects were labelled as diabetic and these subjects were regularly exposed to bursts of OCS therapy for treatment of frequent episodes of asthma exacerbations, making it likely that there was an increased availability of glucose in the airways.

*Moraxella catarrhalis* RDSs were associated with an enrichment of similar pathways related to the innate immune system as for *Haemophilus influenzae* RDSs, but with the additional activation of granulocyte and macrophage colony stimulating factors, confirming previously reported associations.^45^, ^46^ Interestingly, *Tropheryma whipplei* RDSs had no association with any activated pathways.

Characterising clusters by relative abundance of species means that other species are lost in consequence. Thus, the species lost in the *Haemophilus‐*dominant C2 cluster include *Rothia mucilaginosa*, which may be anti‐inflammatory, primarily through inhibition of NF‐κB pathway,^47^ or bioprotective as its levels were diminished in children at risk of asthma,^48^ which may help *Haemophilus influenzae* to promote airway inflammation^49^ through the inhibition of other protective or anti‐inflammatory species. Little is known about the other bacterial species that were also reduced in abundance in the C2 cluster, and also about their clinical significance.

We were interested in understanding the samples with relative dominant species and their significance in terms of the host response. We opted to use an extremely low cut‐off for Shannon's α‐diversity, which has been linked to increased disease severity. Having determined the samples with RDSs, we then clustered on the basis of the relative abundance of the dominant species to find out whether any bacterial species was particularly associated with host response indicating bacterial‐host interactions. This approach we have taken, different from just using the relative abundance directly, has ensured that the microbiome of those with high disease severity would be under focus. Indeed, we found that *Haemophilus influenzae* was most prominent in relative abundance with those with very high relative abundance associated with activated NETosis, IL‐6 trans‐signalling pathway and neutrophil activation, indicating a potential host response to the relative high abundance of *Haemophilus influenzae*.

One of the limitations of the study is the relatively low numbers of samples that constituted an RDS, which is partly due to the stringency of the RDS definition we used, but we managed to obtain the definition of subgroups of *Haemophilus influenzae* RDSs. The *Haemophilus influenzae* RDSs, in particular, will need to be confirmed in larger cohorts of severe asthma by using either quantitative PCR methods or by culture methods. Currently, we are not aware of any metagenomic data available in asthma cohorts that we can use for validation of these findings. There were even lower numbers of severe asthma participants at the one‐year follow‐up that showed that the species RDS profile changes with even non‐RDSs later acquiring RDS status in up to 46% of the severe asthmatics. The factors that determine the stability or change in bacterial species abundance will need to be determined. We also recognise that the metagenomic sequencing used here generate compositional datasets reflecting the proportion of counts per feature per sample, such that relative abundance of species is only available. Therefore, the concept of an RDS must be seen as the *relative* outgrowth of a species within a sample. Finally, another limitation is the lack of information regarding recent antibiotic use. However, all asthma participants were studies at least 6 weeks of any exacerbations when it would have been likely to have been administered an antibiotic, that would have perturbed the sputum microbiome.

Although *Haemophilus influenzae* has been implicated as being an important potential pathogenic bacterial species in asthma and other airways diseases,^4^, ^6^, ^7^ we have delineated precisely for the first time, a restricted phenotype of high relative abundance of *Haemophilus influenzae* in severe asthma associated with high neutrophil inflammation, an adaptive response of NETosis and IL‐6 transsignalling activation. The clinical implications of the definition of this cluster are that such patients with a relative high abundance of *Haemophilus influenzae* associated with a neutrophilic inflammation may potentially respond to *Haemophilus influenzae* vaccine or targeted antibiotic therapy.

## AUTHOR CONTRIBUTIONS

IMA, JR and KFC conceived the idea; IMA, KFC and RD obtained the funding for U‐BIOPRED project; SB, JR and PH obtained the funding for the metagenomic analysis; SB, JR, PH, IMA, MIA, SHC, AV and KFC discussed the approach to data analysis; AV, AA, FXI, MIA and NZK analysed the data; AV, AA and KFC wrote the manuscript; MIA, A‐HMZ and SED contributed to its finalisation and all authors agreed with the final version for submission. All authors gave final approval of the manuscript, had full access to all the data in the study, and had final responsibility for the decision to submit for publication.

## CONFLICT OF INTEREST STATEMENT

Mr Versi has nothing to declare. Dr Azim reports employment through AstraZeneca. Dr Chotirmall has received lecture fees from Chiesi Farmaceutici and AstraZeneca, serves on advisory boards for Boehringer‐Ingelheim, CSL Behring and Pneumagen Ltd. and is on Data and Safety Monitoring Boards (DSMB) for Inovio Pharmaceuticals all outside of the submitted work. Dr Maitland‐van der Zee has received grants from Health Holland and she is the PI of a P4O2 (Precision Medicine for more Oxygen) public private partnership sponsored by Health Holland involving many private partners that contribute in cash and/or in kind (Boehringer Ingelheim, Breathomix, Fluidda, Ortec Logiqcare, Philips, Quantib‐U, Smartfish, SODAQ, Thirona, TopMD and Novartis), received unrestricted research grants from GSK, Boehringer Ingelheim and Vertex, received consulting fees paid to her institution from Boehringer Ingelheim and AstraZeneca, and received honoraria for lectures paid to her institution from GlaxoSmithKline; outside the submitted work. Dr. Dahlén reports personal fees from AZ, Cayman Chemicals, GSK, Novartis, Regeneron, Sanofi, TEVA, outside the submitted work. Dr Chung has received honoraria for participating in Advisory Board meetings of Roche, Merck, Shionogi and Rickett‐Beckinson and has also been remunerated for speaking engagements for Novartis and AZ. Dr Riley worked for and had shares in GSK. Dr. Bates reports to be currently an employee of Johnson & Johnson and to have previously worked and holds stock in GSK. Dr Uddin is an employee and holds shares in AstraZeneca. Dr Djukanovic declares consulting fees from Synairgen, Sanofi and Galapagos, lecture fees from GSK, AZ and Airways Vista and he holds shares from Synairgen. Dr Howarth is an employee of GSK. Dr Montuschi, Dr Kermani, Dr Adcock, Dr Ivan and Dr Abdel‐Aziz have nothing to declare.

## ETHICS STATEMENT

This study was approved by teh Ethics Commmittee of each participating clinical institution. All patients gave written informed consent to participate in the study.

## Supporting information

Supporting Information

## Data Availability

The metagenomic sequence data have been submitted to the NCBI under accession number PRJNA946921 and are accessible at the following link: https://www.ncbi.nlm.nih.gov/sra/PRJNA946921.
