# Supplementary material for: A severe asthma phenotype of excessive airway Haemophilus influenzae relative abundance associated with sputum neutrophilia
Source: Clin Transl Med. 2024 Aug 26;14(9):e70007. doi: 10.1002/ctm2.70007 (PMC11347389; doi:10.1002/ctm2.70007)
Supplement: Supplementary file 1 — Supporting Information [file CTM2-14-e70007-s001.docx]

*Supplementary file*

**A severe asthma phenotype of excessive airway**

***Haemophilus influenzae* relative abundance associated with sputum neutrophilia**

Ali Versi, Adnan Azim, Fransiskus Xaverius Ivan, Mahmoud I Abdel-Aziz, Stewart Bates, John Riley, Mohib Uddin, Nazanin Zounemat Kermani, Anke-Hilse Maitland-Van Der Zee, Sven-Eric Dahlen, Ratko Djukanovic, Sanjay H Chotirmall, Peter Howarth, Ian M Adcock, Kian Fan Chung on behalf of the U-BIOPRED study group^¶^

**Contents**

1.Method for designating species relative dominant species (RDS)

2.Summary of DNA extraction, sequencing and quality control

3. Definition of relative abundance of bacterial species in sputum

4. Functional metagenomic pipeline using HUMAnN2

5. References

6. Supplementary Figures S1 to S13

7. Supplementary Tables S1 to S5

8. U-BIOPRED Consortium project team

1. **Method for designating species relative dominant species (RDS)**

The Shannon’s diversity index is described by the following equation:


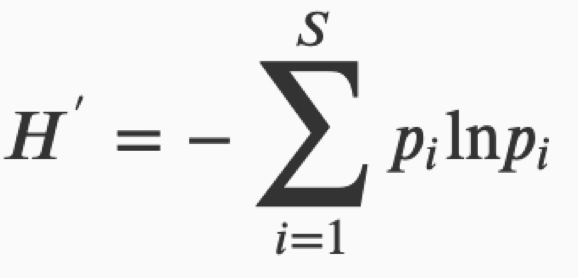


where p*_i_* denotes the proportion of the entire sample composed of species *i (1).*

The Shannon’s diversity index has origins in information theory and is used to measure entropy. The equation denotes the principle that as the more taxa or species there are and as their equalness in abundance increases, then there is more difficulty associated with predicating the next taxa/species and its abundance - hence there is higher entropy or disorder in the sample (2). Thus, the Shannon’s α diversity in the current context is a measure of richness and evenness of the species in a given sample. The term -p_i_ln(p_i_) provides the evenness contribution to the equation.

Therefore, as the Shannon’s diversity decreases, the evenness and richness of species within the sample also decreases. A decrease in evenness of abundance within a sample denotes that the abundance of one or more species rises above the rest of the sample. To determine the correct point at which when the evenness is reduced enough to be abnormal, a 2-standard deviation below the mean of the mild-moderate asthma and healthy control (MMA/HC) group was used as an arbitrary threshold. Applying this threshold to the Severe Asthma group allowed us to identify the species which are attributed to this low evenness within the samples. Those samples within severe asthma that do not fall below this threshold would be considered not to have severe asthma associated with any degree of microbial dysbiosis.

This method allows us to identify the species which are associated with this reduction in evenness within the sample and can then be defined as a relative dominant bacterial species (RDS). These samples with a particular RDS species can then be looked at with paired transcriptomics data to gain insight into the host response to the bacterial species relative dominance. This then allows for an analysis into the heterogeneity of the host response to a particular species.

Because the interest of this study was to better understand how low diversity severe asthma is abnormal, the healthy control (HC) subjects and the mild-moderate asthma (MMA) subjects were combined together to form the one comparator group. This is reasonable to combine the 2 groups because the mean α diversity of MMA and HC were within 5% of each other with the standard deviation of MMA cohort less than the standard deviation of the HC cohort. The Z-test between the two distributions of MMA vs HC showed they were not significantly different, with a Z-statistic of -0.3, suggesting the samples were of the same distribution and a non-significant p-value (0.76). The distribution plots of the two cohorts is shown in Supplementary Fig S3A below.

1. **DNA extraction, sequencing and quality control**

Sputum sampling induction was carried out by participants inhaling nebulised hypertonic saline solutions from participants, as previously described (3). Sputum plugs were isolated and frozen immediately at -70°C. Genomic DNA was extracted from the frozen samples using MoBio Tissue and Cells DNA Isolation Kit (Qiagen, Germantown, USA) at 250μl input volume. Negative and positive controls were used for each batch and the extracted DNA was stored at -20°C. DNA samples were prepared for sequencing with the Illumina Nextera kit (Illumina, San Diego, USA) and quantified with Quant-iT dsDNA High Sensitivity assays (ThermoFisher Scientific, Waltham, USA). Libraries were pooled and run with 100 base-pair paired-end sequencing protocols on the Illumina HiSeq 2500 platform. FastQC version 0.11.8 (4) was used to assess the quality of the metagenomic sequencing reads. Host reads were removed using bowtie2 version 2.3.5.1 (with its default parameters) (5) to align the quality-filtered reads to the human reference genome (hg38) and samtools version 1.9 (6) was used to remove the aligned host-reads. Following removal of host reads, the median (± IQR) depth of sequencing reads was 1,580,827 (± 1,169,351) reads; the lowest and the highest were 426,428 and 11,393,132 respectively.

Shotgun metagenomic sequencing involves the random fragmentation of all DNA present in a given sample, followed by the sequencing of these fragments. This approach addresses the limitations of amplicon sequencing which targets specific genetic markers only rather than giving an unbiased comprehensive view.

**3. Definition of relative abundance of bacterial species in sputum**

We used the MetaPhlAn2 pipeline (7) to profile the microbial composition of each sample to a species level resolution to derive the relative abundance of each species from shotgun sequencing. MetaPhlAn2 employs approximately 1 million unique clade-specific marker genes, which are genes that are highly conserved among specific taxonomic groups, from 13,500 bacterial and archaeal reference genomes. The pipeline counts the number of sequences for each sample which maps to each marker gene and uses these counts to estimate the relative abundance of each species using total sum normalisation.

**4. Functional metagenomic pipeline using HUMAnN2**

We used the HUMAnN2 pipeline (8) to profile the metabolic pathway content contributed by known and uncharacterized species within a given microbiome sample from shotgun metagenomics. This pipeline uses a tiered search strategy comprised of three search phases. The first tier finds species within the sample and then constructs a custom gene database based on these species from functionally-annotated pangenomes. In the second tier the entrie sample is aligned to the gene database constructed in the first tier resulting in gene alignment statistics per species in addition to unmapped reads. The final tier these unmapped reads are aligned to databases such as UniProt (and UniPathway) (9,10). The alignment statistics are then stratified into per-species and unclassified contributions and gene abundance values are applied to construct and quantify metabolic pathway prevalence.

**5. References**

1. Shannon CE. A Mathematical Theory of Communication. Bell Syst Tech J. 1948;27(3):379–423.

2. Willis AD. Rarefaction, Alpha Diversity, and Statistics. Front Microbiol [Internet]. 2019 [cited 2023 Jun 28];10. Available from: https://www.frontiersin.org/articles/10.3389/fmicb.2019.02407

3. Versi A, Ivan FX, Abdel-Aziz MI, Bates S, Riley J, Baribaud F, et al. Haemophilus influenzae and Moraxella catarrhalis in sputum of severe asthma with inflammasome and neutrophil activation. Allergy. 2023 Jun 7;

4. Andrew S. FastQC: A Quality Control Tool for High Throughput Sequence Data [Online. 2010.

5. Langmead B, Salzberg SL. Fast gapped-read alignment with Bowtie 2. Nat Methods. 2012;9(4):357–9.

6. Li H, Handsaker B, Wysoker A, Fennell T, Ruan J, Homer N. The Sequence Alignment/Map format and SAMtools. Vol. 25. Bioinformatics (Oxford, England; 2009. p. 2078–9.

7. Truong DT, Franzosa EA, Tickle TL, Scholz M, Weingart G, Pasolli E. MetaPhlAn2 for enhanced metagenomic taxonomic profiling. Nat Methods. 2015;12(10):902–3.

8. Franzosa EA, McIver LJ, Rahnavard G, Thompson LR, Schirmer M, Weingart G, et al. Species-level functional profiling of metagenomes and metatranscriptomes. Nat Methods. 2018 Nov;15(11):962–8.

9. UniProt: a hub for protein information. Nucleic Acids Res. 2015 Jan 28;43(Database issue):D204–12.

10. Morgat A, Coissac E, Coudert E, Axelsen KB, Keller G, Bairoch A. UniPathway: a resource for the exploration and annotation of metabolic pathways. Nucleic Acids Res. 2012;40(Database issue).

**Supplementary Figure S1**

| **Algorithm for RDS determination** | | | | |
| --- | --- | --- | --- | --- |
|  | ***Input****: abundance_matrix, Shannon_threshold* | | | |
|  | ***N <- input.length*** | | | |
|  | ***For species = 0 to N:*** | | | |
|  |  | ***For cutoff = 0 to 75 with step 0.5:*** | | |
|  |  |  | ***new_abundance_matrix <- select_samples_of _species_less_than(species, cutoff)*** | |
|  |  |  | ***If mean_shannon_diversity(new_abundance_matrix) >= shannon_threshold:*** | |
|  |  |  |  | ***Output <- [species, cutoff]*** |
|  |  |  |  | ***break*** |
|  |  | ***End*** | | |
|  | ***End*** | | | |

Algorithm of deriving Relative Dominant Species using pseudo-code.

**Supplementary Figure S2.**


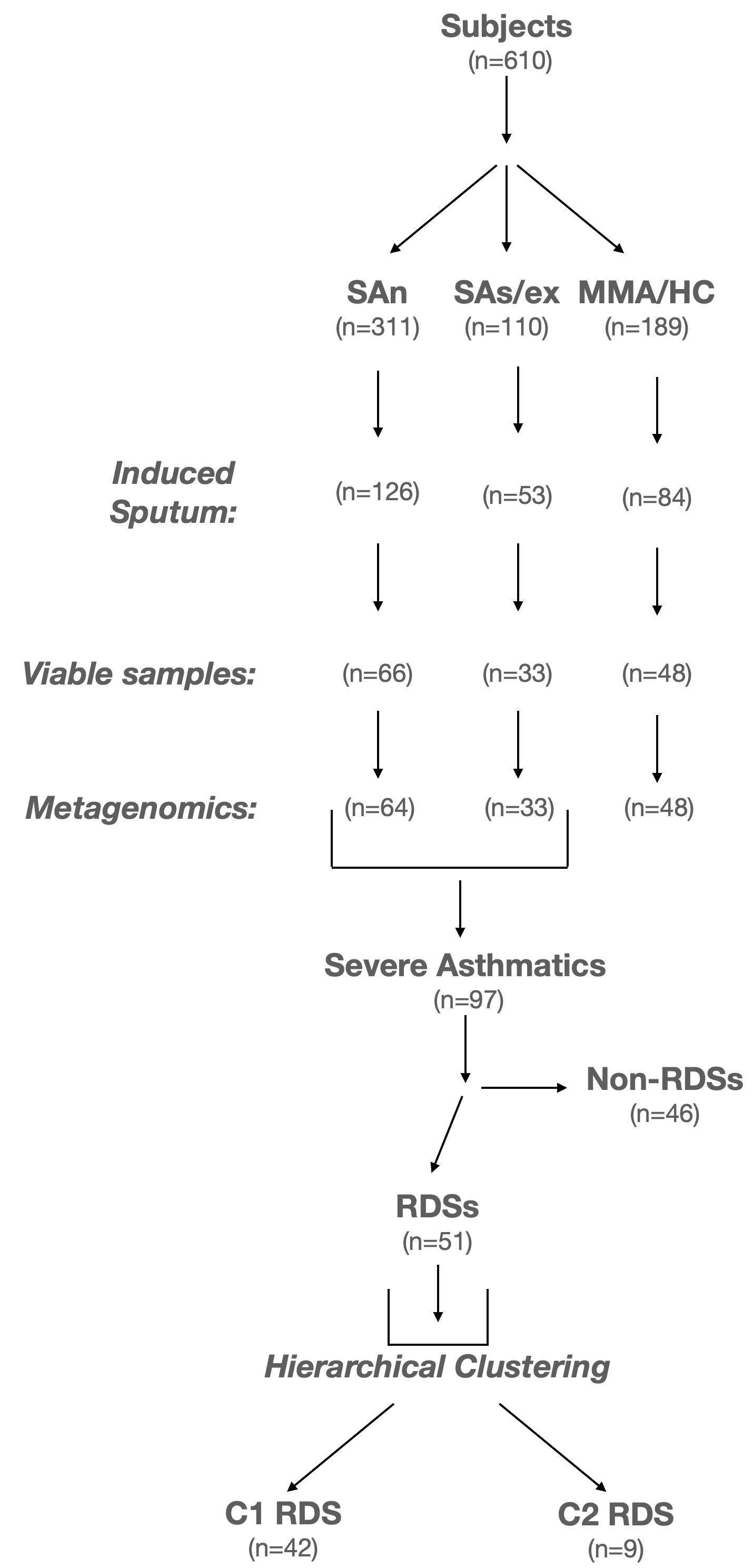


Consort diagram depicting the repartition of asthma subjects recruited in U-BIOPRED cohort into various groups and in the hierarchical clustering with 3 clusters of RDS.

Hi: *Haemophilus influenzae*; MMA/HC: mild-moderate asthma and Healthy control subjects; RDS: relative dominant species; SAn: severe non-smoking asthma; SAs/ex: severe ex-smoking or current smoking asthma.

**Supplementary Figure S3.**

**A**


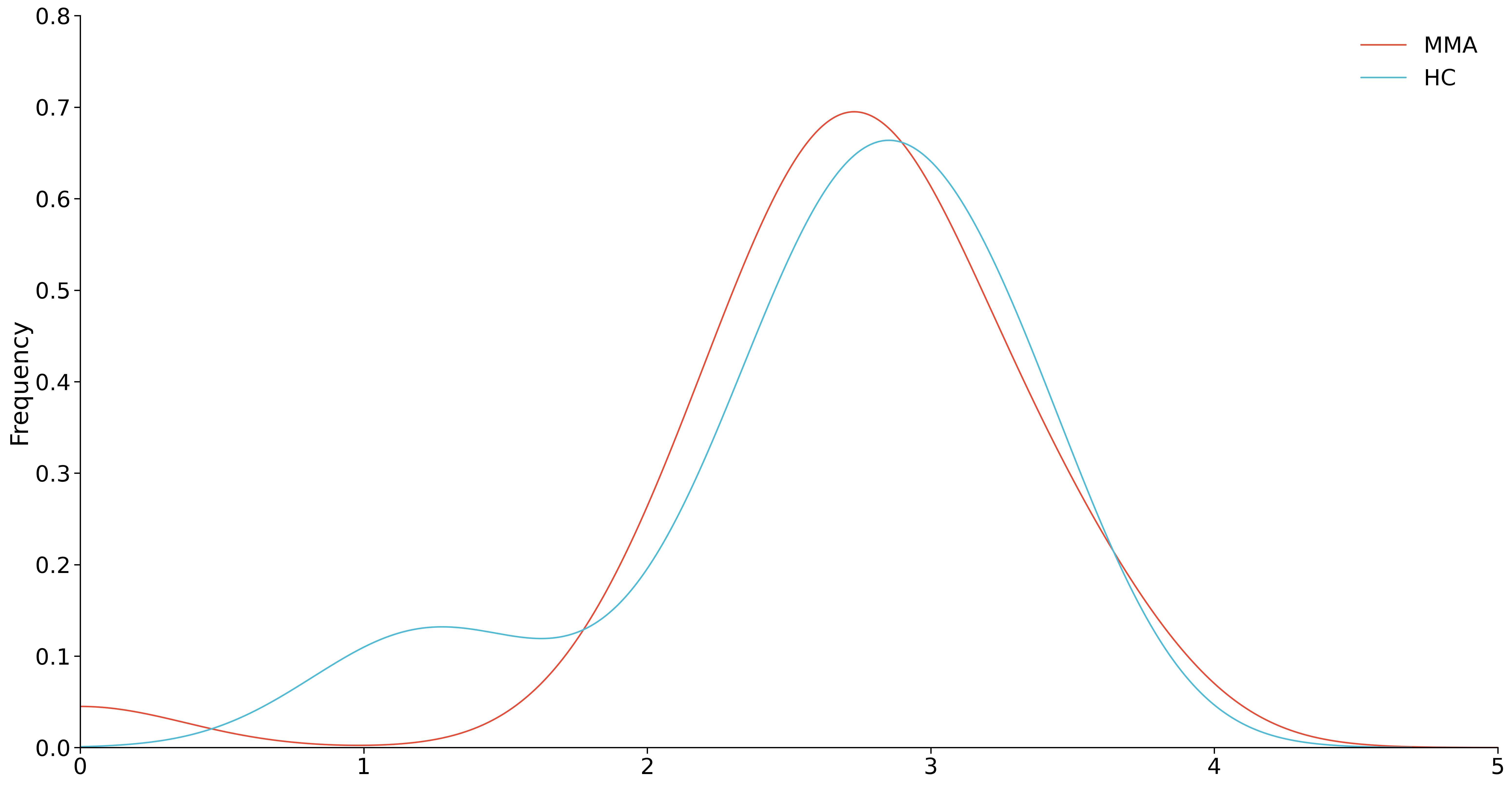


Shannon’s α-diversity

**B**

**
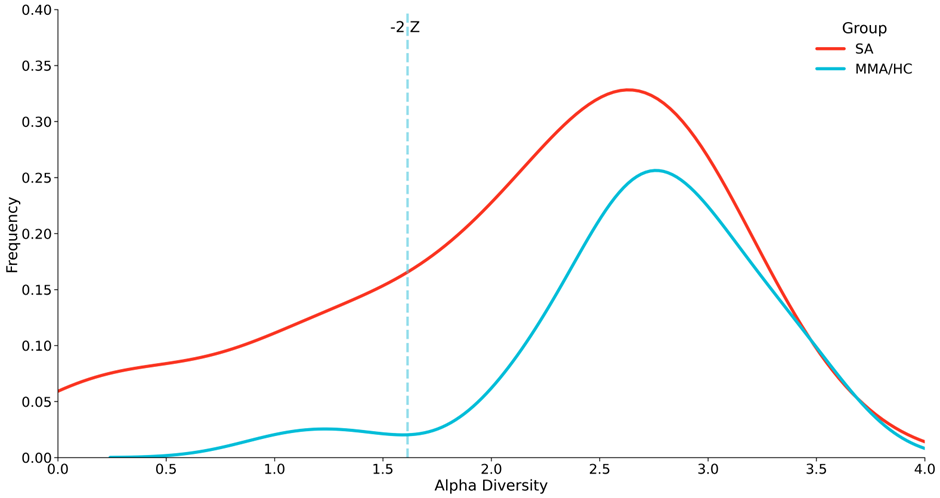
**

Shannon’s α-diversity

1. Mean **Shannon’s α-diversity** distribution of mild-moderate asthma (MMA) and healthy controls (HC) were within 5% of each other with the standard deviation of MMA cohort less than the standard deviation of the HC cohort.
2. **Relative dominant species (RDS) cut-off methodology.** The Shannon’s α-diversity distribution of combined mild-moderate asthma/healthy controls (MMA/HC) and severe asthma (SA). Those with α-diversity in the range of or less than –2-Z score were included as RDSs.

**Supplementary Figure S4.**

**(A)**

**
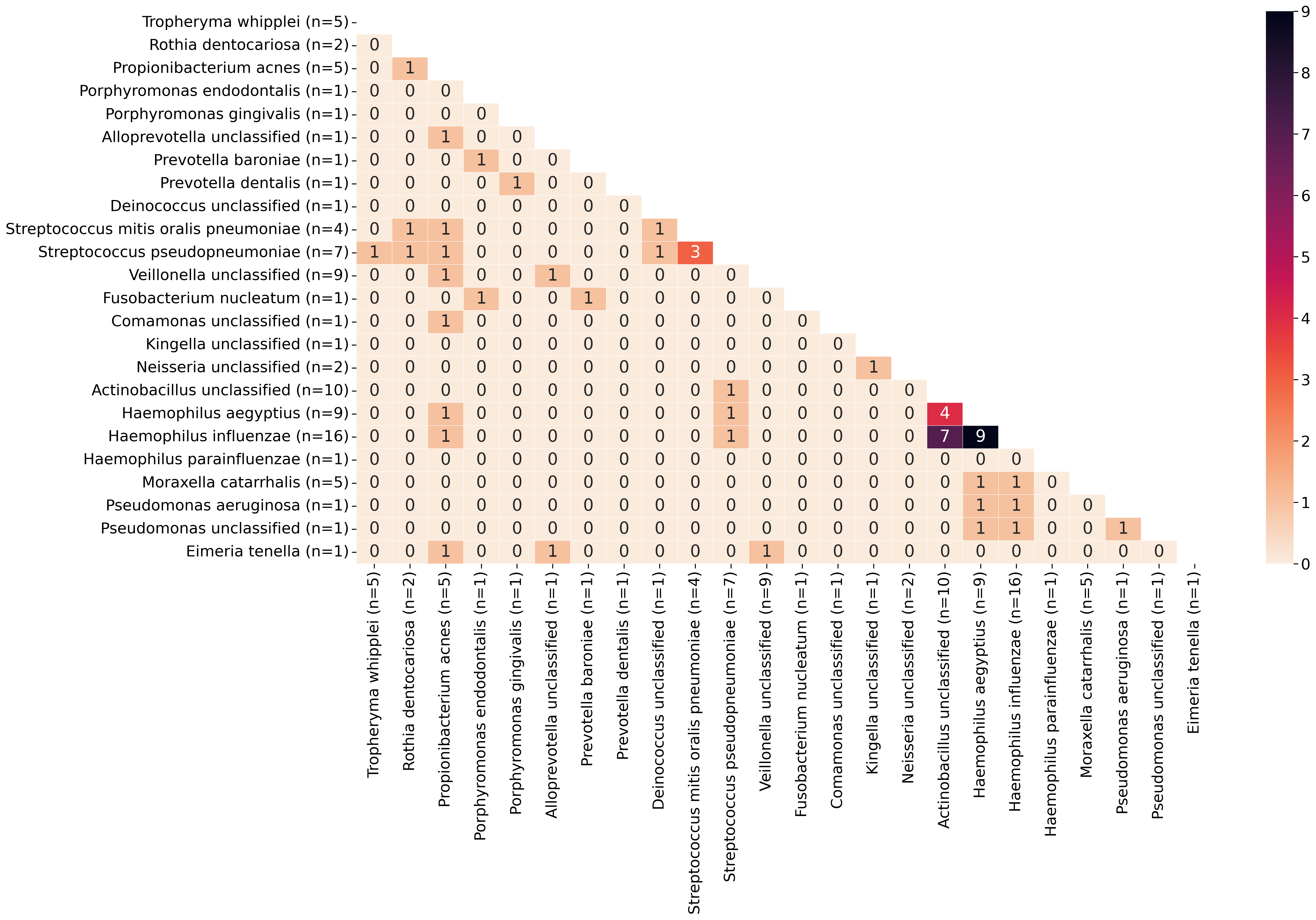
**

**(B)**

**
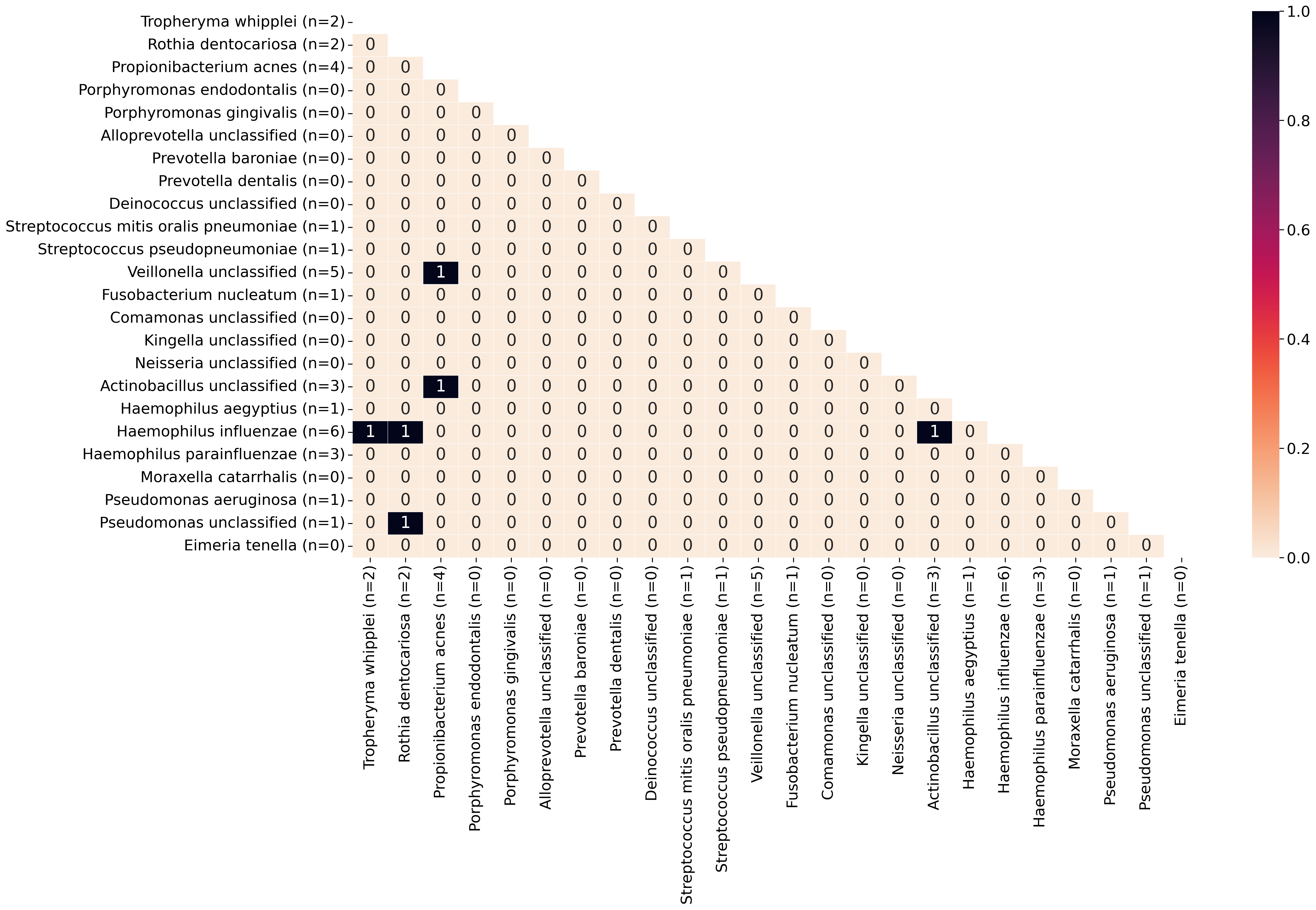
**

**Relative dominant species (RDS) co-occurrence heatmap.** The heatmap shows the co-occurence of RDSs within severe asthma groups (combined SAn and SAs/ex). The heatmap defines the number of samples containing more than one RDS type. The number of samples containing a particular RDS is denoted in the labels of the plot for each RDS. **Panel A** is for baseline and **Panel B** is for follow-up.

**Supplementary Figure S5**

**
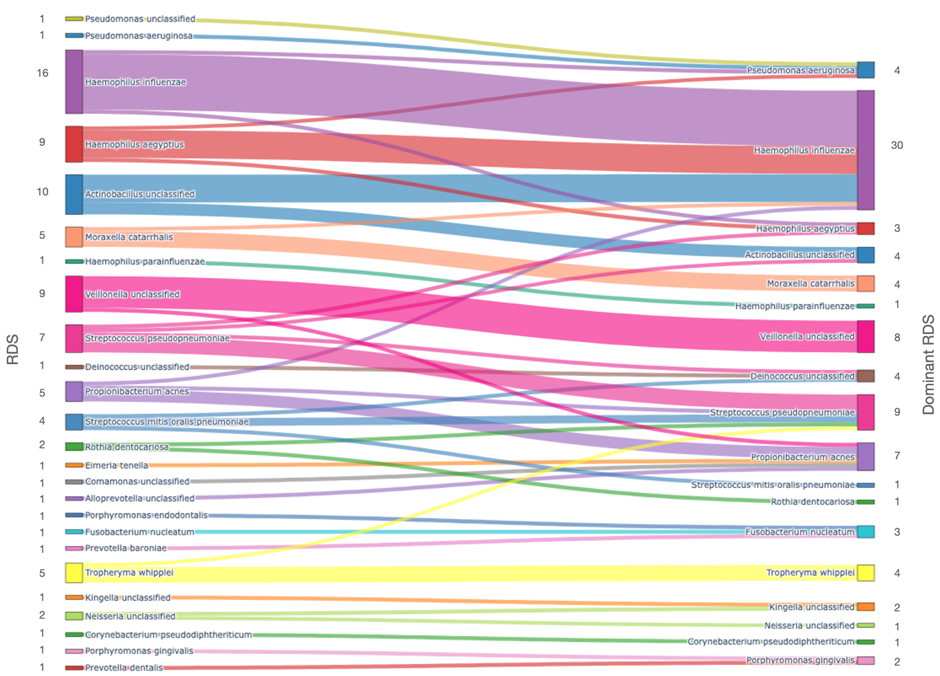
**

**Sankey plot** showing the number of RDSs (samples may contain more than one RDS) on left-hand column to dominant RDS categorization (in samples that have one dominant RDS) on the right hand column.

**Supplementary Figure S6.**

**A B**

**
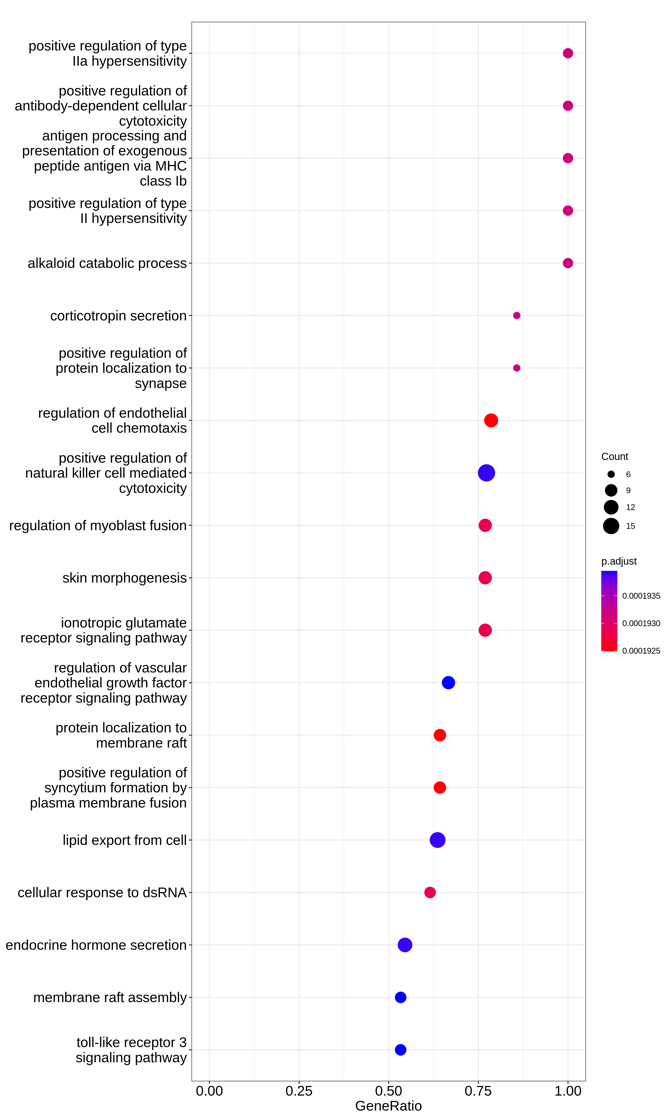

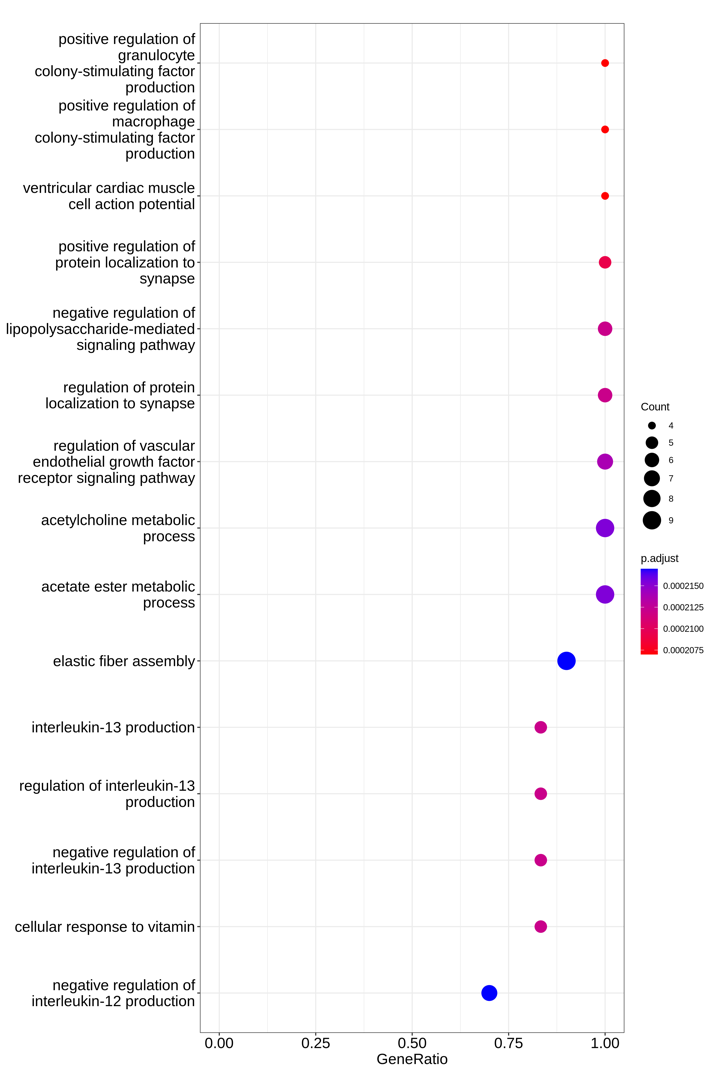
**

GO enrichment dot-plots between the *Haemophilus influenzae* (panel A) and *Moraxella catarrhalis* bacterial relative dominance (panel B) compared to MMA group.

**Supplementary Figure S7**

**
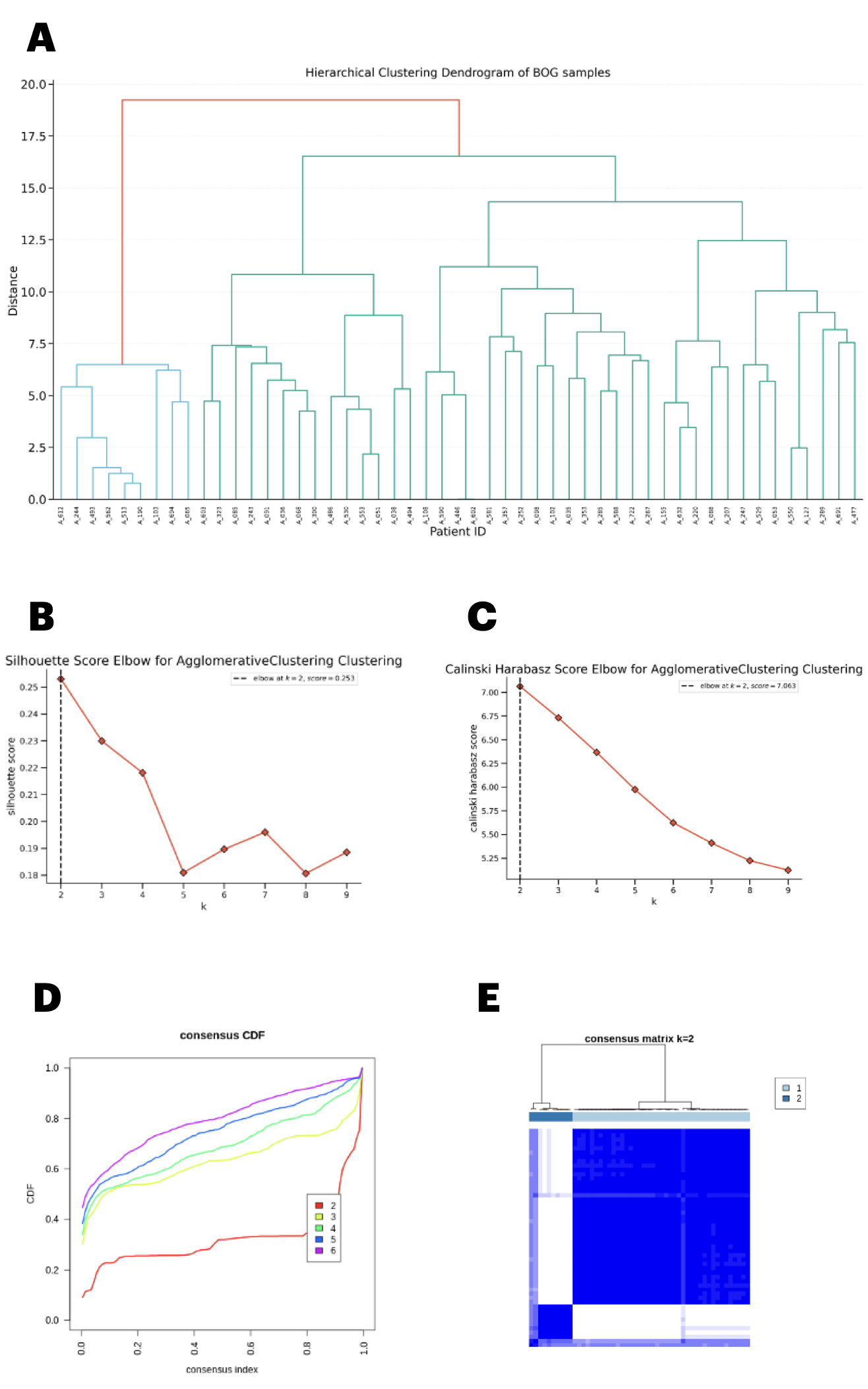
**

**Supplementary Metagenomic hierarchical clustering of sputum metagenomic species-level abundance of bacterial RDS samples (k=2).** (A) Dendrogram of the hierarchical clustering. (B) Silhouette score. (C) Calinski-Harabasz score (D) CDF plot displaying consensus distributions for each number of clusters (*k*). (E) Consensus matrix heat map depicting consensus values on a white to blue color scale of cluster for κ=2

**
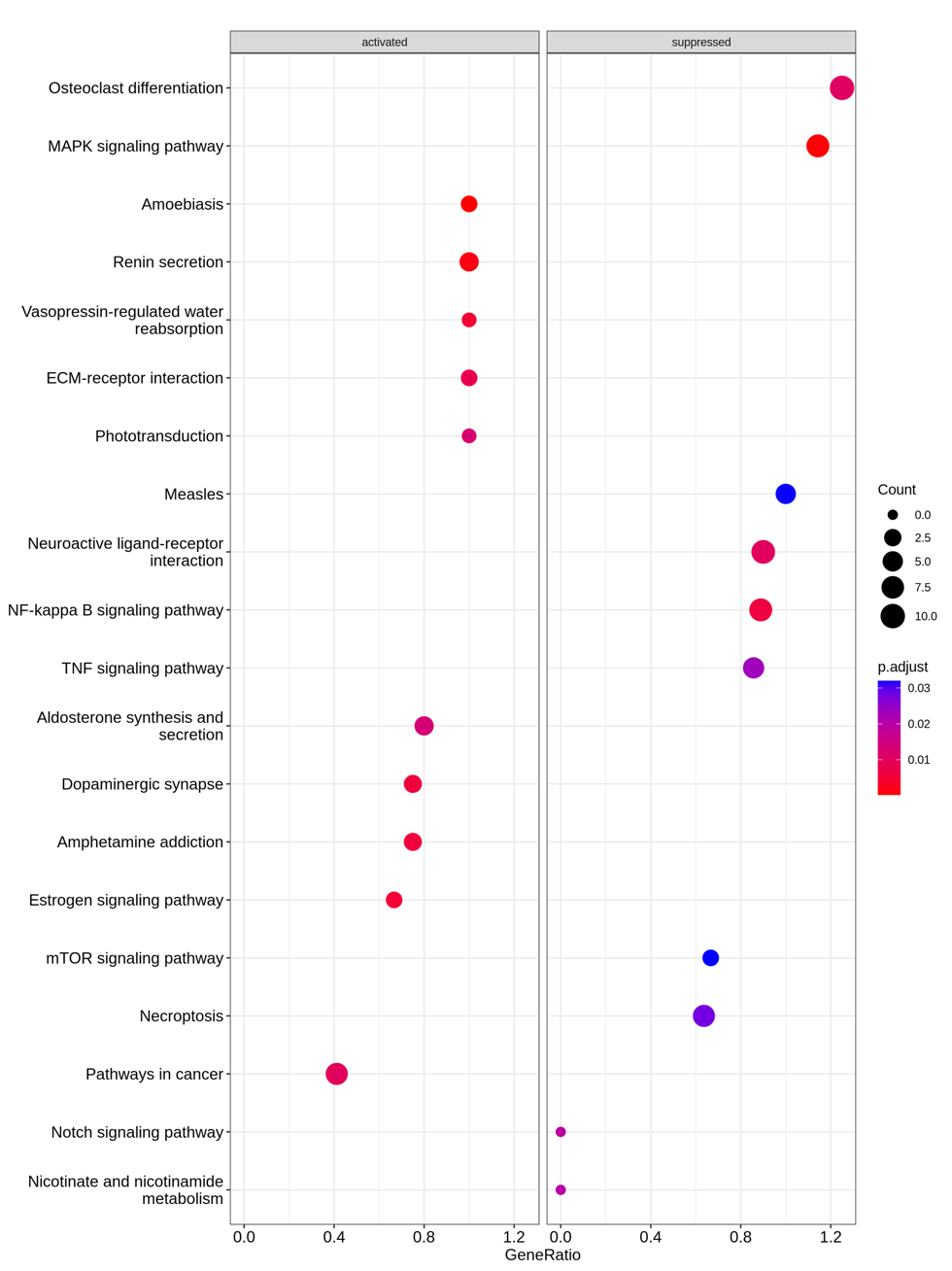
Supplementary Figure S8.**

**
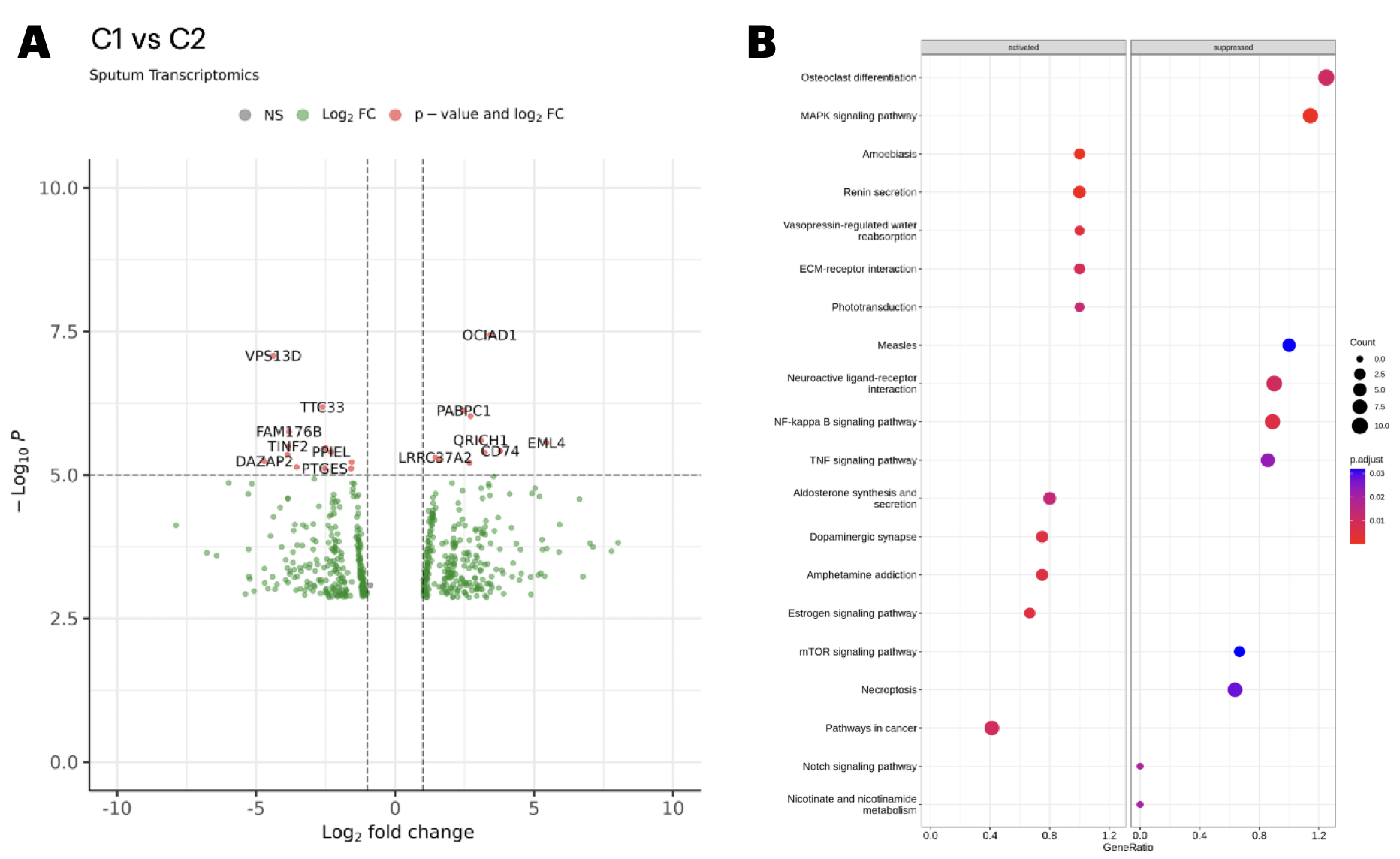
**

**Panel A**. DEG and GO pathway enrichment between C1 and C2 clusters.

**Panel B.** Volcano plot depicts the sputum transcriptomics DEG between C1 and C2 cluster. Dot-plots depict the GO pathway enrichment for C1 and C2 cluster.

**Supplementary Figure S9.**





**Gene set variation analysis (GSVA) enrichment score** between the C1 and C2 and the Non- RDS**.** Boxplot of the enrichment scores of 6 gene signatures representing Netosis (1), Blood Eosinophil (2), IL6-transignalling (3), Oxidative phosphorylation (Oxphos) (4), Innate lymphoid cell type 2 (ILC2) (5), Lung-Tissue Resident Macrophage (Macrophage TR) (6), IL13-T2 (7), Neutrophil (8) and Th17 (9). GSVA was performed in R using the Bioconductor GSVA package to estimate the variation in gene signature enrichment scores (ES).

Mann–Whitney *U* test was used for comparison between groups.

Significance: *:*P*≤0.05.

**Supplementary Figure S10. ANCOM-BC plot depicting differentially prevalent microbial pathways** **between C2 and C1*.*** The blue labels indicate a positive log-fold change and the red labels indicate a negative log-fold change.

**
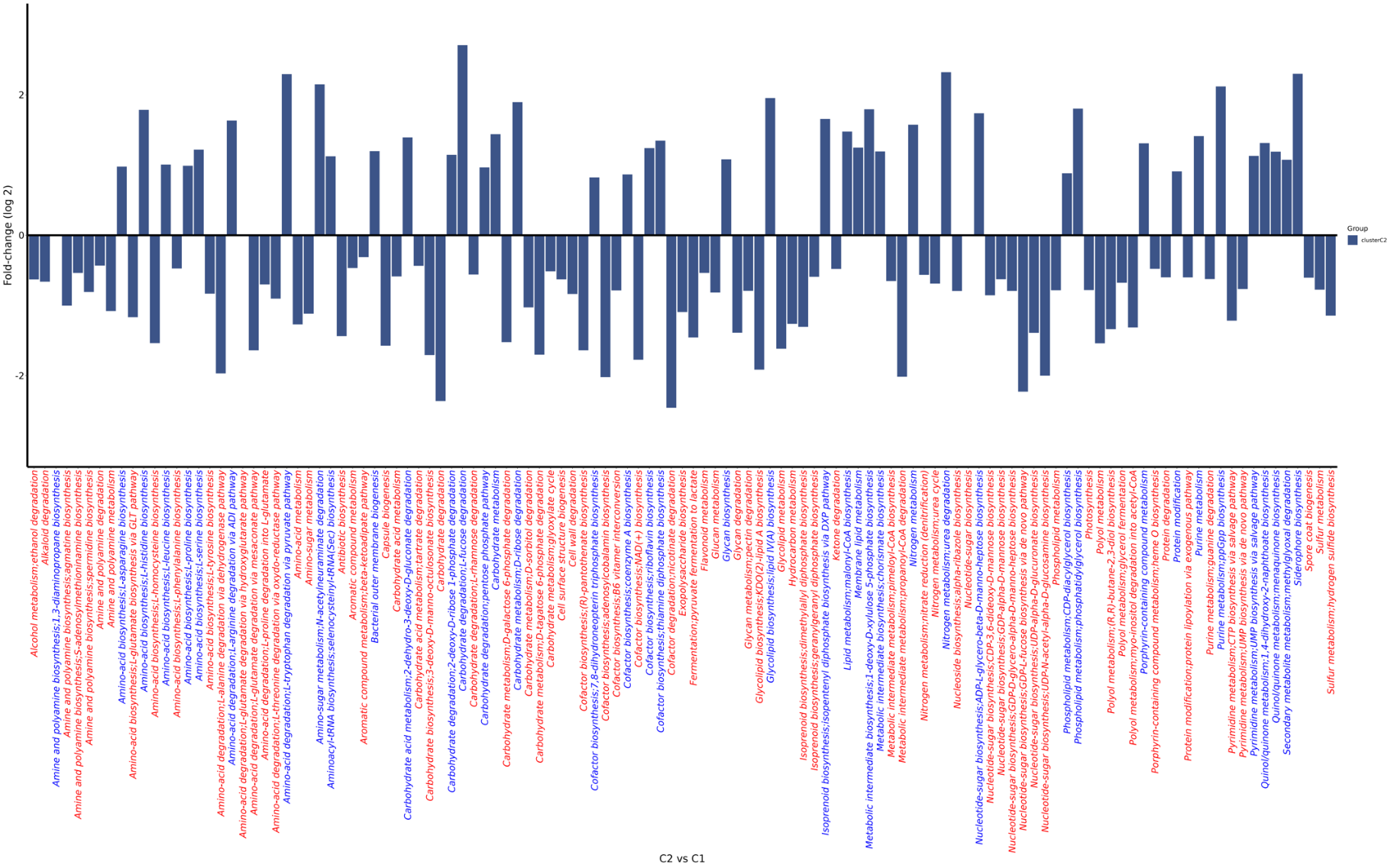
**

**Supplementary Figure S11.**


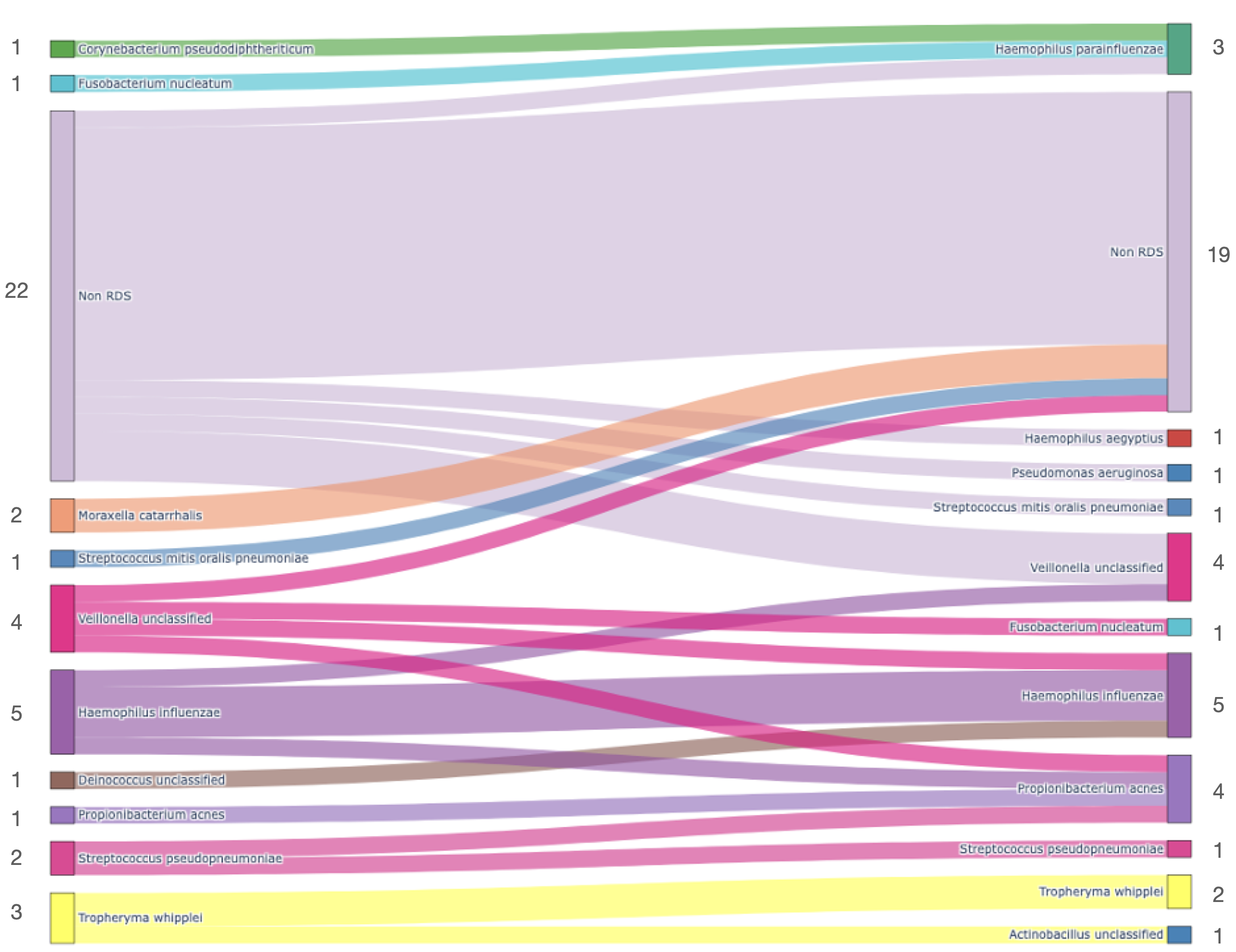


**Sankey plot** depicting the stability of the dominant RDS within each sample at baseline and follow-up. The number of samples for the dominant RDSs and Non-RDS groups (n=43 in total) at baseline and at one year follow-up is shown alongside each of the 2 time-points with regards to the bacterial species.

**Supplementary Figure S12**


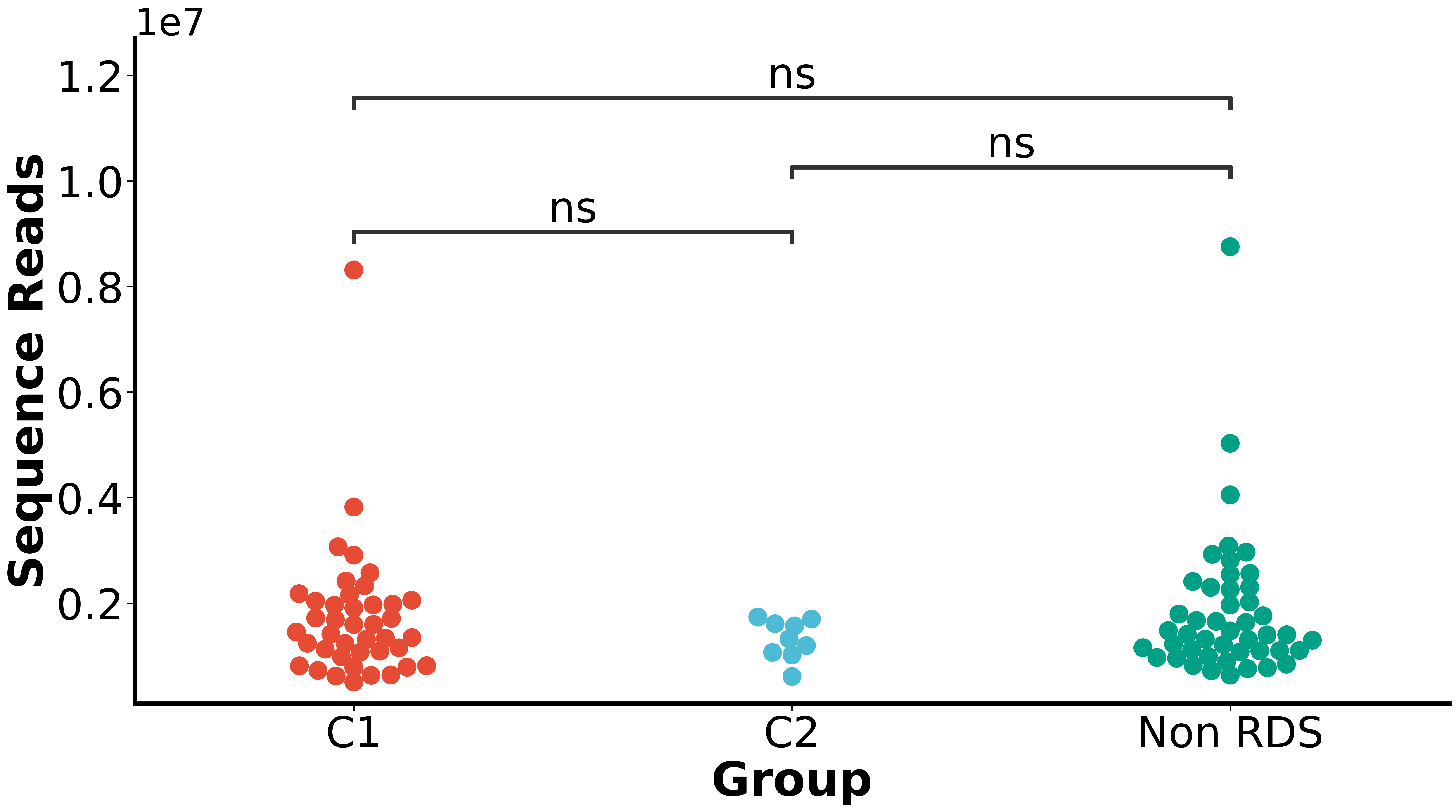


**Beeswarm plot** depicting the number of non-host reads for each sample using the clusters C1 and C2 and the group, Non-RDS. Mann–Whitney *U* test was used for comparison between groups. ns: not significant.

**Supplementary Figure S13**


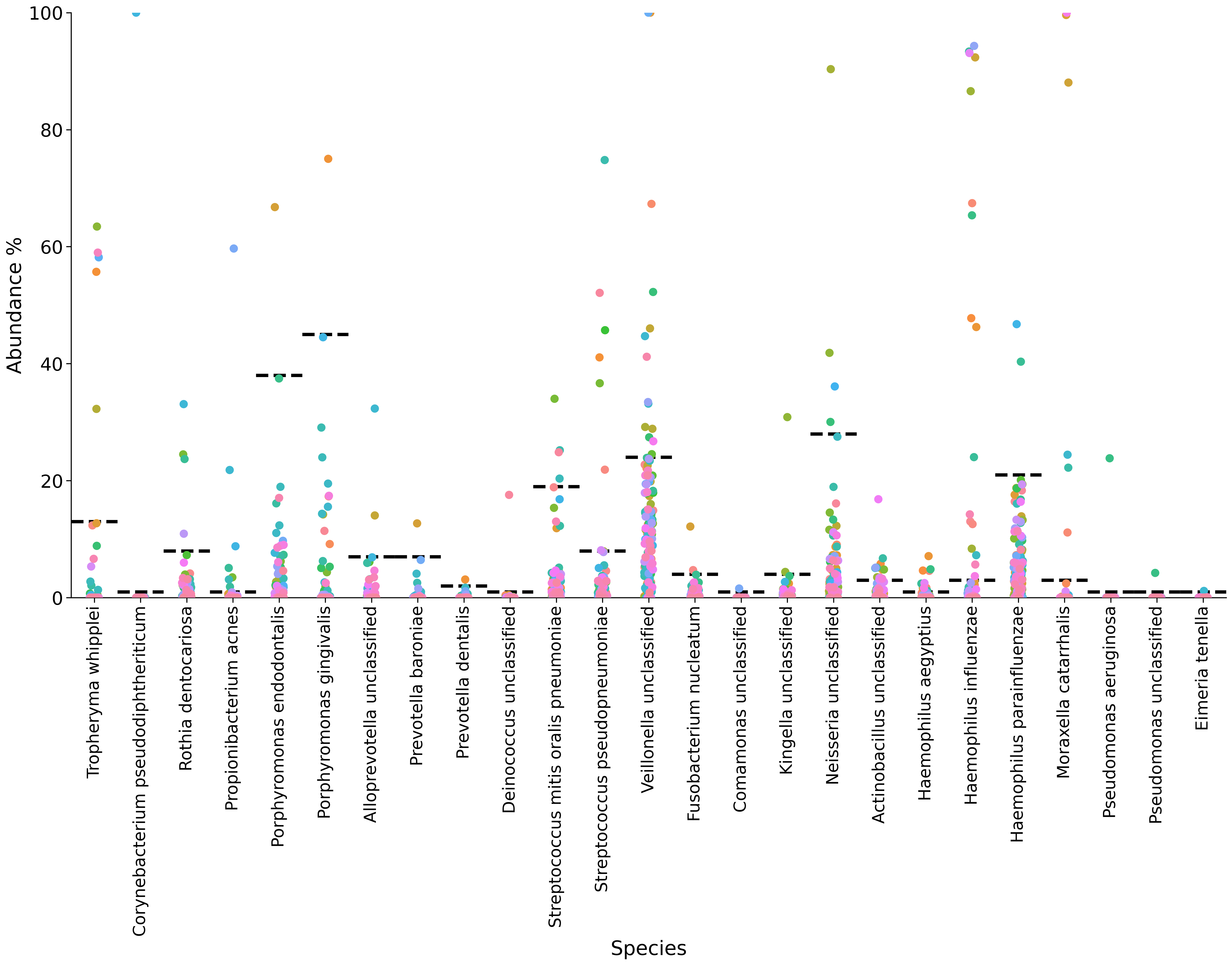


**Dot blot** depicting the abundance of each RDS species for all baseline samples. The black dotted line shows the cut-off of abundance of that species becoming an RDS.

**References:**

1. de Bont CM, Boelens WC, Pruijn GJM. NETosis, complement, and coagulation: a triangular relationship. Cell Mol Immunol. 2019 Jan;16(1):19–27.

2. Barnig C, Alsaleh G, Jung N, Dembélé D, Paul N, Poirot A, et al. Circulating Human Eosinophils Share a Similar Transcriptional Profile in Asthma and Other Hypereosinophilic Disorders. PLOS ONE. 2015 Nov 2;10(11):e0141740.

3. Jevnikar Z, Östling J, Ax E, Calvén J, Thörn K, Israelsson E, et al. Epithelial IL-6 trans-signaling defines a new asthma phenotype with increased airway inflammation. J Allergy Clin Immunol. 2019 Feb;143(2):577–90.

4. Liberzon A, Subramanian A, Pinchback R, Thorvaldsdóttir H, Tamayo P, Mesirov JP. Molecular signatures database (MSigDB) 3.0. Bioinforma Oxf Engl. 2011 Jun 15;27(12):1739–40.

5. Björklund ÅK, Forkel M, Picelli S, Konya V, Theorell J, Friberg D, et al. The heterogeneity of human CD127(+) innate lymphoid cells revealed by single-cell RNA sequencing. Nat Immunol. 2016 Apr;17(4):451–60.

6. Misharin AV, Morales-Nebreda L, Reyfman PA, Cuda CM, Walter JM, McQuattie-Pimentel AC, et al. Monocyte-derived alveolar macrophages drive lung fibrosis and persist in the lung over the life span. J Exp Med. 2017 Aug 7;214(8):2387–404.

7. Alevy YG, Patel AC, Romero AG, Patel DA, Tucker J, Roswit WT, et al. IL-13–induced airway mucus production is attenuated by MAPK13 inhibition. J Clin Invest. 2012 Dec 3;122(12):4555–68.

8. Abbas AR, Baldwin D, Ma Y, Ouyang W, Gurney A, Martin F, et al. Immune response in silico (IRIS): immune-specific genes identified from a compendium of microarray expression data. Genes Immun. 2005 Jun;6(4):319–31.

9. Zhang H, Nestor CE, Zhao S, Lentini A, Bohle B, Benson M, et al. Profiling of human CD4+ T-cell subsets identifies the TH2-specific noncoding RNA GATA3-AS1. J Allergy Clin Immunol. 2013 Oct;132(4):1005–8.

**Supplementary Table S1. Characteristics of participants by asthma severity.**

|  | **Baseline** | | | |  | **Longitudinal** |
| --- | --- | --- | --- | --- | --- | --- |
|  | **SA** | **MMA** | **HC** | **P Value** | ^#^**P value** | **SA** |
| Subjects, n | 97 | 25 | 23 | NA | NA | 43 |
| Age (years) | 55.0 [46.0,62.0] | 45.0 [28.0,51.0] | 41.0 [27.5,49.0] | <0.001 | NS | 56.5 [51.2,62.2] |
| Female, n (%) | 56 (57.7) | 12 (48.0) | 7 (30.4) | NS | NS | 25 (56.8) |
| BMI | 28.7 (5.7) | 25.6 (4.8) | 25.5 (3.2) | <0.01 | NS | 27.9 (4.9) |
| Duration years, | 25.3 (16.2) | 23.5 (16.6) | N/A | NS | NA | 25.3 (16.4) |
| Current smoking, n (%) | 11 (11.3) | 0 | 0 | <0.001 | NS | 4 (9.1) |
| Oral corticosteroid daily, n (%) | 39 (40.2) | 17 (37.0) | 0 | <0.001 | NS | 12 (27.3) |
| ACQ-5 score, | 2.3 (1.2) | 1.1 (0.8) | 0.1 (0.2) | <0.001 | <0.001 | 2.0 (1.3) |
| Exacerbations, per year, | 2.2 (2.1) | 0.6 (1.0) | 0.0 | <0.001 | <0.01 | 2.0 (2.1) |
| Nasal polyposis, n (%) | 33 (34.0) | 4 (16.0) | 2 (8.7) | NS | NS | 16 (36.4) |
| Eczema, n (%) | 25 (25.8) | 9 (36.0) | 0 | <0.05 | <0.005 | 11 (25.0) |
| Allergic rhinitis, n (%) | 36 (37.1) | 13 (52.0) | 2 (8.7) | <0.005 | <0.005 | 16 (36.4) |
| GERD, n (%) | 42 (43.3) | 3 (12.0) | 1 (4.3) | <0.001 | NS | 20 (45.5) |
| Blood Eosinophils μL^-1^ | 0.3 (0.3) | 0.2 (0.1) | 0.1 (0.1) | <0.001 | <0.01 | 0.3 (0.2) |
| Blood Neutrophils μL^-1^ | 5.4 (2.5) | 3.7 (1.2) | 3.5 (1.4) | <0.001 | NS | 5.4 (2.5) |
| Sputum Neutrophils (%) | 56.9 (25.3) | 48.0 (24.5) | 42.6 (28.0) | <0.05 | NS | 61.1 (22.4) |
| Sputum Eosinophils (%) | 13.8 (20.3) | 3.8 (11.1) | 0.3 (0.5) | <0.01 | NS | 11.1 (18.0) |
| Sputum Lymphocytes (%) | 1.3 (1.3) | 2.3 (2.5) | 1.4 (1.1) | <0.05 | NS | 1.5 (1.7) |
| Sputum Macrophage (%) | 27.9 (21.7) | 46.5 (23.9) | 55.7 (27.8) | <0.001 | NS | 26.1 (19.1) |
| FEV1% predicted | 64.8 (20.2) | 90.5 (19.0) | 104.1 (11.8) | <0.001 | 0.005 | 62.1 (16.7) |
| FeNO (ppb), | 37.4 (36.6) | 39.6 (32.7) | 20.4 (9.6) | NS | <0.01 | 40.1 (37.4) |
| Smoking (pack years) | 16.7 (17.1) | 3.4 (1.5) | 3.0 (2.8) | NS | NS | 16.0 (16.6) |
| Antibiotic current, n (%) | 17 (17.5) | 0 | 0 | <0.001 | NS | 7 (15.9) |
| Serum CRP (mg/L) | 6.6 (13.6) | 1.6 (2.3) | 1.0 (0.9) | <0.05 | NS | 6.6 (15.2) |
| Serum IL1α (pg/ml) | 35.4 (7.2) | 31.9 (6.6) | 32.7 (3.5) | <0.05 | NS | 36.0 (8.4) |
| Serum IL6 (pg/ml) | 1.3 (1.0) | 0.7 (0.7) | 0.5 (0.3) | <0.001 | NS | 1.1 (0.7) |
| Serum IL8 (pg/ml) | 4.4 (5.5) | 3.5 (1.4) | 2.9 (1.7) | NS | NS | 5.5 (7.9) |
| Serum C5a (pg/ml) | 45.5 (25.4) | 34.4 (18.5) | 33.1 (21.3) | <0.05 | NS | 47.8 (28.9) |

**Abbreviations:**

ACQ5: Asthma Control Questionnaire score; BMI: Body mass index; CRP: Serum C-Reactive Protein; FeNO: Fractional exhaled nitric oxide; FEV1: Forced Expiratory Volume in the first second pre-salbutamol; FVC: Forced Vital Capacity; GERD: Gastroesophageal reflux disease; N/A: Not applicable; NS: Not significant; ppb: parts per billion. Data shown as mean (Standard Deviation) unless variable is categorical where n (%). Age shown as median [95% Confidence intervals]. P value: comparing SA (severe asthmatics), MMA (mild-moderate asthmatics) & HC (Healthy Controls).

P value: comparing the three groups: SA, MMA, HC.

^#^P value: comparing the two groups: MMA, HC.

**Supplementary Table S2. Characteristics of Relative Dominant Species (RDS) and non-RDSs.**

|  | **Baseline** | | | **Longitudinal** | | |
| --- | --- | --- | --- | --- | --- | --- |
|  | **RDSs** | **Non-RDSs** | **P Value** | **RDSs** | **Non-RDSs** | **P Value** |
| Subjects, n | 51 | 46 | NA | 24 | 19 |  |
| Age (years) | 54.0 [47.0,59.5] | 55.0 [45.2,62.8] | NS | 56.0 [46.8,61.2] | 58.0 [53.5,65.5] | NS |
| Female, n (%) | 27 (52.9) | 29 (63.0) | NS | 12 (50.0) | 12 (63.2) | NS |
| BMI | 28.6 (5.1) | 28.8 (6.3) | NS | 27.8 (4.9) | 28.1 (5.1) | NS |
| Duration years, | 27.9 (16.1) | 22.4 (16.1) | NS | 28.9 (15.2) | 21.5 (17.4) | NS |
| Current smoking, n (%) | 5 (9.8) | 6 (13.0) | NS | 2 (8.3) | 1 (5.3) | NS |
| Oral corticosteroid daily, n (%) | 22 (43.1) | 17 (37.0) | NS | 8 (33.3) | 4 (21.1) | NS |
| ACQ-5 score, | 2.3 (1.2) | 2.3 (1.2) | NS | 2.1 (1.3) | 1.8 (1.2) | NS |
| Exacerbations, per year, | 2.1 (2.2) | 2.4 (2.1) | NS | 1.5 (1.8) | 2.3 (2.3) | NS |
| Nasal polyposis, n (%) | 18 (35.3) | 15 (32.6) | NS | 7 (29.2) | 9 (47.4) | NS |
| Eczema, n (%) | 14 (27.5) | 11 (23.9) | NS | 7 (29.2) | 3 (15.8) | NS |
| Allergic rhinitis, n (%) | 25 (49.0) | 11 (23.9) | <0.05 | 10 (40.0) | 6 (31.6) | NS |
| GERD, n (%) | 24 (47.1) | 18 (39.1) | NS | 12 (48.0) | 8 (42.1) | NS |
| Blood Eosinophils μL^-1^ | 0.3 (0.3) | 0.3 (0.3) | NS | 0.2 (0.1) | 0.4 (0.2) | <0.005 |
| Blood Neutrophils μL^-1^ | 5.7 (2.5) | 5.1 (2.6) | NS | 6.0 (2.8) | 4.7 (1.9) | NS |
| Sputum Neutrophils (%) | 63.6 (24.7) | 49.5 (24.0) | 0.005 | 65.8 (20.5) | 55.1 (23.7) | NS |
| Sputum Eosinophils (%) | 14.5 (20.2) | 13.1 (20.5) | NS | 8.1 (13.6) | 14.9 (22.2) | NS |
| Sputum Lymphocytes (%) | 1.0 (1.1) | 1.6 (1.4) | <0.05 | 1.3 (1.7) | 1.9 (1.5) | NS |
| Sputum Macrophage (%) | 20.8 (18.5) | 35.7 (22.5) | 0.001 | 24.5 (20.7) | 28.1 (17.2) | NS |
| FEV1% predicted | 62.4 (21.3) | 67.4 (18.7) | NS | 59.9 (17.5) | 64.9 (15.6) | NS |
| FeNO (ppb), | 35.0 (34.9) | 40.1 (38.6) | NS | 31.5 (26.9) | 50.9 (46.0) | NS |
| Smoking (pack years) | 15.3 (16.1) | 18.3 (18.4) | NS | 17.4 (19.3) | 12.5 (6.6) | NS |
| Antibiotic current, n (%) | 7 (13.7) | 10 (21.7) | NS | 4 (16.0) | 3 (15.8) | NS |
| Serum CRP (mg/L) | 6.6 (12.2) | 6.6 (15.3) | NS | 6.4 (11.2) | 6.8 (19.5) | NS |
| Serum IL1α (pg/ml) | 35.7 (7.9) | 35.1 (6.4) | NS | 36.5 (9.4) | 35.2 (7.1) | NS |
| Serum IL6 (pg/ml) | 1.4 (1.2) | 1.1 (0.8) | NS | 1.0 (0.7) | 1.2 (0.7) | NS |
| Serum IL8 (pg/ml) | 4.8 (7.0) | 4.0 (3.0) | NS | 6.0 (10.2) | 4.9 (4.3) | NS |
| Serum C5a (pg/ml) | 50.4 (28.8) | 39.8 (19.6) | <0.05 | 54.8 (32.8) | 38.9 (20.5) | NS |

**Abbreviations:**

ACQ5: Asthma Control Questionnaire score; BMI: Body mass index; CRP: Serum C-Reactive Protein; FeNO: Fractional exhaled nitric oxide; FEV1: Forced Expiratory Volume in the first second pre-salbutamol; FVC: Forced Vital Capacity; GERD: Gastroesophageal reflux disease; N/A: Not applicable; NS: Not significant; ppb: parts per billion. Data shown as mean (Standard Deviation) unless variable is categorical where n (%). Age shown as median [95% Confidence intervals]. P value: comparing RDSs and Non-RDSs.

**Supplementary Table S3. P-Values of severe asthma with *Haemophilus influenzae (Hi), Moraxella catarrhalis (Mc)* & *Tropheryma whipplei (Tw)* relative dominant species (RDS) compared with Non-RDS corresponding to Table 2.**

|  | **Hi RDS vs Non-RDS P Value** | **Mc RDS vs Non-RDS P Value** | **Tw RDS vs Non-RDS P Value** |
| --- | --- | --- | --- |
| Subjects, n | NA | NA | NA |
| Age (years) | NS | NS | NS |
| Females, n (%) | NS | NS | NS |
| BMI | <0.05 | NS | NS |
| Duration years, | <0.001 | NA | NA |
| Current Smoking, n (%) | NS | NS | NS |
| Oral corticosteroid daily, n (%) | NS | NS | NS |
| ACQ-5 score, | NS | NS | NS |
| Exacerbations, per year, | NS | <0.05 | NS |
| Nasal polyposis, n (%) | <0.05 | NS | NS |
| Eczema, n (%) | NS | NS | NS |
| Allergic rhinitis, n (%) | NS | NS | NS |
| GERD, n (%) | NS | NS | NS |
| Blood Eosinophils μL^-1^ | NS | NS | NS |
| Blood Neutrophils μL^-1^ | NS | NS | NS |
| Sputum Neutrophils (%), | <0.05 | NS | NS |
| Sputum Eosinophils (%) | NS | <0.001 | NS |
| Sputum Lymphocytes (%) | <0.005 | <0.05 | NS |
| Sputum Macrophage (%) | <0.001 | NS | NS |
| FEV1 (% predicted) | NS | NS | <0.001 |
| FeNO (ppb) | NS | NS | NS |
| Smoking pack years | NS | NS | NS |
| Antibiotic current, n (%) | NA | NA | NS |
| CRP (mg/L) | NS | NS | NS |
| IL-1α (pg/ml) | NS | NS | NS |
| IL-6 (pg/ml) | NS | NS | NS |
| Serum IL-8 (pg/ml) | NS | NS | NS |
| Serum C5a (pg/ml) | <0.05 | NS | NS |

Only samples with no co-occurring BOGs of the three species (Hi, Mc, Tw) were selected.

**Abbreviations:** ACQ5: Asthma Control Questionnaire score; BMI: Body mass index; CRP: Serum C-Reactive Protein; FeNO: Fractional exhaled nitric oxide; FEV1: Forced Expiratory Volume in the first second pre-salbutamol; FVC: Forced Vital Capacity; GERD: Gastroesophageal reflux disease; MMA/HC: Mild-moderate asthma/Healthy controls; N/A: Not applicable; NS: Not significant; ppb: parts per billion. Data shown as mean (Standard Deviation) unless variable is categorical where n (%). Age shown as median [95% Confidence intervals].

Hi – Haemophilus influenzae, Mc- *Moraxella catarrhalis* and Tw - *Tropheryma whipplei* RDS group

**Table S4. Characteristics of severe asthma with Actinobacillus unclassified, Streptococcus pseudopneumoniae & Veillonella unclassified relative dominant species (RDS).**

|  | ***Actinobacillus***  ***unclassified* RDS** | ***Streptococcus***  ***pseudopneumoniae***  **RDS** | ***Veillonella***  ***unclassified***  **RDS** | **non-RDSs** | **P Value** | ^#^**P value** | ^##^**P Value** |
| --- | --- | --- | --- | --- | --- | --- | --- |
| Subjects, n | 2 | 4 | 9 | 46 | N/A | N/A | N/A |
| Age (years) | 58.5 [55.2,61.8] | 54.5 [51.5,56.5] | 55.0 [50.0,58.0] | 55.0 [45.2,62.8] | NS | NS | NS |
| Females, n (%) | 2 (100.0) | 2 (50.0) | 5 (55.6) | 29 (63.0) | NS | NS | NS |
| BMI | 33.7 (0.4) | 29.5 (5.5) | 27.8 (5.5) | 28.8 (6.3) | <0.001 | NS | NS |
| Duration years, | 9.5 (4.9) | 32.2 (16.3) | 20.2 (10.8) | 22.4 (16.1) | NS | NS | NS |
| Current Smoking, n (%) | 0 (0) | 2 (50.0) | 1 (11.1) | 6 (13.0) | NS | NS | NS |
| Oral corticosteroid daily, n (%) | 2 (100.0) | 3 (75.0) | 3 (33.3) | 17 (37.0) | NS | NS | NS |
| ACQ-5 score, | 2.2 (1.1) | 2.9 (1.6) | 1.6 (1.2) | 2.3 (1.2) | NS | NS | NS |
| Exacerbations, per year, | 2.0 (1.4) | 2.2 (3.2) | 1.9 (1.3) | 2.4 (2.1) | NS | NS | NS |
| Nasal polyposis, n (%) | 1 (50.0) | 1 (25.0) | 4 (44.4) | 15 (32.6) | NS | NS | NS |
| Eczema, n (%) | 0 (0) | 0 (0) | 1 (11.1) | 11 (23.9) | NS | NS | NS |
| Allergic rhinitis, n (%) | 2 (100.0) | 2 (50.0) | 3 (33.3) | 11 (23.9) | NS | NS | NS |
| GERD, n (%) | 2 (100.0) | 1 (25.0) | 4 (44.4) | 18 (39.1) | NS | NS | NS |
| Blood Eosinophils μL^-1^ | 0.8 (0.4) | 0.3 (0.1) | 0.4 (0.3) | 0.3 (0.3) | NS | NS | NS |
| Blood Neutrophils μL^-1^ | 8.8 (2.3) | 4.5 (1.9) | 4.8 (1.5) | 5.1 (2.6) | NS | NS | NS |
| Sputum Neutrophils (%), | 59.8 (9.0) | 67.3 (19.4) | 49.2 (24.5) | 49.5 (24.0) | NS | NS | NS |
| Sputum Eosinophils (%) | 27.1 (9.0) | 3.0 (1.5) | 14.4 (18.8) | 13.1 (20.5) | NS | <0.005 | NS |
| Sputum Lymphocytes (%) | 0.4 (0.5) | 1.9 (1.1) | 1.1 (0.8) | 1.6 (1.4) | NS | NS | NS |
| Sputum Macrophage (%) | 12.8 (0.5) | 27.9 (18.6) | 35.4 (26.3) | 35.7 (22.5) | <0.001 | NS | NS |
| FEV1 (% predicted) | 60.2 (3.2) | 63.9 (25.4) | 67.1 (15.1) | 67.4 (18.7) | NS | NS | NS |
| FeNO (ppb) | 34.0 (26.9) | 47.2 (55.3) | 45.6 (47.0) | 40.1 (38.6) | NS | NS | NS |
| Smoking pack years | 2.0 (0.8) | 21.9 (19.8) | 15.8 (14.7) | 18.3 (18.4) | <0.001 | NS | NS |
| Antibiotic current, n (%) | 0 (0) | 0 (0) | 1 (11.1) | 10 (21.7) | NS | NS | NS |
| CRP (mg/L) | 25.5 (19.8) | 3.9 (4.3) | 1.9 (2.2) | 6.6 (15.3) | NS | NS | NS |
| IL-1α (pg/ml) | 30.8 (2.3) | 38.2 (10.2) | 36.4 (4.7) | 35.1 (6.4) | NS | NS | NS |
| IL-6 (pg/ml) | 5.0 (1.9) | 1.3 (0.7) | 1.0 (0.5) | 1.1 (0.8) | NS | NS | NS |
| Serum IL-8 (pg/ml) | 4.7 (0.3) | 3.8 (0.9) | 3.8 (2.3) | 4.0 (3.0) | NS | NS | NS |
| Serum C5a (pg/ml) | 52.8 (2.1) | 44.1 (12.3) | 46.3 (13.0) | 39.8 (19.6) | 0.001 | NS | NS |

Only samples with no co-occurring RDSs of the five species were selected.

**Abbreviations:** ACQ5: Asthma Control Questionnaire score; BMI: Body mass index; CRP: Serum C-Reactive Protein; FeNO: Fractional exhaled nitric oxide; FEV1: Forced Expiratory Volume in the first second pre-salbutamol; FVC: Forced Vital Capacity; GERD: Gastroesophageal reflux disease; MMA: Mild-moderate asthma; N/A: Not applicable; NS: Not significant; ppb: parts per billion. Data shown as mean (Standard Deviation) unless variable is categorical where n (%). Age shown as median [95% Confidence intervals].

P value: comparing RDSs and non-RDSs.

P value: comparing the five groups: **Actinobacillus RDS** and non-RDSs.

^#^P value: comparing the four groups: **Streptococcus RDS** and non-RDSs.

^##^P value: comparing the three cluster groups: **Veillonella RDS** and non-RDSs.

**Supplementary Table S5. Abundance of RDS species present in C1 and C2 clusters and in Non-RDS**

| **Bacterial species BOG** | **C1 (n=42)** | **C2 (n=9)** | **Non-RDS (n=46)** |
| --- | --- | --- | --- |
| *Tropheryma whipplei* | 7.06 ± 17.92 (R=5) | 0.00 ± 0.00 (R=0) | 0.29 ± 1.88 |
| *Corynebacterium pseudodiphtheriticum* | 2.38 ± 15.43 (R=1) | 0.00 ± 0.00 (R=0) | 0.00 ± 0.00 |
| *Rothia dentocariosa* | 1.70 ± 6.26 (R=2) | 0.09 ± 0.22 (R=0) | 0.81 ± 1.50 |
| *Propionibacterium acnes* | 2.39 ± 9.77 (R=5) | 0.07 ± 0.21 (R=0) | 0.01 ± 0.06 |
| *Porphyromonas endodontal* | 2.27 ± 10.33 (R=1) | 0.00 ± 0.00 (R=0) | 2.34 ± 6.09 |
| *Porphyromonas gingival* | 3.86 ± 13.79 (R=1) | 0.00 ± 0.00 (R=0) | 2.15 ± 5.79 |
| *Alloprevotella unclassified* | 1.01 ± 5.00 (R=1) | 0.00 ± 0.00 (R=0) | 0.59 ± 1.63 |
| *Prevotella baroniae* | 0.32 ± 1.96 (RR=1) | 0.00 ± 0.00 (R=0) | 0.20 ± 0.97 |
| *Prevotella dentalis* | 0.09 ± 0.48 (RR=1) | 0.00 ± 0.00 (R=0) | 0.07 ± 0.20 |
| *Deinococcus unclassified* | 0.42 ± 2.71 (RR=1) | 0.00 ± 0.00 (R=0) | 0.03 ± 0.09 |
| *Streptococcus pneumoniae* | 4.29 ± 8.47 (RR=4) | 0.51 ± 1.08 (R=0) | 1.52 ± 2.16 |
| *Streptococcus pseudopneumoniae* | 7.46 ± 16.97 (RR=7) | 0.00 ± 0.00 (R=0) | 0.57 ± 1.03 |
| *Veillonella unclassified* | 15.47 ± 23.76 (RR=9) | 1.53 ± 3.22 (R=0) | 11.46 ± 6.50 |
| *Fusobacterium nucleatum* | 0.41 ± 1.90 (RR=1) | 0.00 ± 0.00 (R=0) | 0.39 ± 0.79 |
| *Comamonas unclassified* | 0.04 ± 0.24 (RR=1) | 0.00 ± 0.00 (R=0) | 0.00 ± 0.00 |
| *Kingella unclassified* | 0.80 ± 4.75 (RR=1) | 0.30 ± 0.80 (R=0) | 0.37 ± 0.75 |
| *Neisseria unclassified* | 4.44 ± 9.50 (RR=2) | 1.66 ± 2.88 (R=0) | 2.15 ± 3.19 |
| *Actinobacillus unclassified* | 0.81 ± 2.75 (RR=4) | 3.68 ± 1.79 (R=6) | 0.54 ± 0.69 |
| *Haemophilus aegyptius* | 0.17 ± 0.79 (RR=2) | 2.62 ± 2.42 (R=7) | 0.07 ± 0.21 |
| *Haemophilus influenzae* | 2.12 ± 5.13 (RR=7) | 76.28 ± 19.92 (R=9) | 0.34 ± 0.73 |
| *Haemophilus parainfluenzae* | 3.94 ± 8.08 (RR=1) | 0.59 ± 1.10 (R=0) | 6.09 ± 4.89 |
| *Moraxella catarrhalis* | 5.58 ± 20.63 (RR=4) | 1.24 ± 3.71 (R=1) | 0.09 ± 0.39 |
| *Pseudomonas aeruginosa* | 0.00 ± 0.00 (RR=0) | 2.65 ± 7.94 (R=1) | 0.00 ± 0.00 |
| *Pseudomonas unclassified* | 0.00 ± 0.00 (RR=0) | 0.47 ± 1.41 (RR=1) | 0.00 ± 0.00 |
| *Eimeria tenella* | 0.03 ± 0.18 (RR=1) | 0.00 ± 0.00 (RR=0) | 0.00 ± 0.00 |

**Abbreviations:**

RDS: Relative dominant species; MMA/HC: Mild-moderate asthma/Healthy controls;. Data shown as mean ± Standard Deviation with number of BOG samples for that species in parenthesis. C1 – Cluster 1 of BOG samples; C2 – Cluster 2 of BOG samples. B denotes the number of samples which contain BOGs for that species. B within brackets indicate number of RDS.

**List of the U-BIOPRED Consortium project team members**

| **U-BIOPRED Supplementary authors** | |
| --- | --- |
| **Name** | **Affiliation** |
| Abdel-Aziz MI | Department of Respiratory Medicine, Amsterdam UMC, University of Amsterdam, Amsterdam, The Netherlands |
| Adcock I M | National Heart and Lung Institute, Imperial College, London, UK; |
| Andersson LI | Department of Respiratory Medicine, Karolinska University Hospital, Stockholm, Sweden |
| Auffray C | European Institute for Systems Biology and Medicine, CNRS-ENS-UCBL-INSERM, Lyon, France; |
| Badi YE, | National Heart and Lung Institute, Imperial College, London, UK; |
| Bakke P | Department of Clinical Science, University of Bergen, Bergen, Norway; |
| Bansal A T | Acclarogen Ltd, St. John’s Innovation Centre, Cambridge, UK; |
| Baribaud F | Janssen R&D, LLC, Spring House, PA, USA |
| Bates S | Respiratory Therapeutic Unit, GSK, London, UK; |
| Bel E H | Academic Medical Centre, University of Amsterdam, Amsterdam, The Netherlands; |
| Bigler J | *Previously Amgen Inc* |
| Billing B | Department of Respiratory Medicine, Karolinska University Hospital, Stockholm, Sweden |
| Bisgaard H | COPSAC, Copenhagen Prospective Studies on Asthma in Childhood, Herlev and Gentofte Hospital,  University of Copenhagen, Copenhagen, Denmark |
| Boedigheimer M J | Amgen Inc.; Thousand Oaks, USA |
| Bønnelykke K | COPSAC, Copenhagen Prospective Studies on Asthma in Childhood, Herlev and Gentofte  Hospital, University of Copenhagen, Copenhagen, Denmark; |
| Brandsma J | University of Southampton, Southampton, UK |
| Brinkman P | Academic Medical Centre, University of Amsterdam, Amsterdam, The Netherlands; |
| Bucchioni E | Chiesi Pharmaceuticals SPA, Parma, Italy |
| Burg D | Centre for Proteomic Research, Institute for Life Sciences, University of Southampton, Southampton, UK |
| Bush A | National Heart and Lung Institute, Imperial College, London, UK; Royal Brompton and Harefield NHS trust, UK |
| Caruso M | Dept. Clinical and Experimental Medicine, University of Catania, Catania, Italy; |
| Chalekis R | Institute of Environmental Medicine, Centre for Allergy Research, Karolinska Institutet, Stockholm, Sweden |
| Chanez P | Assistance publique des Hôpitaux de Marseille - Clinique des bronches, allergies et sommeil, Aix Marseille Université, Marseille, France |
| Chung K F | National Heart and Lung Institute, Imperial College, London, UK; |
| Checa T | Institute of Environmental Medicine, Centre for Allergy Research, Karolinska Institutet, Stockholm, Sweden |
| Compton C H | Respiratory Therapeutic Unit, GSK, London, UK |
| Corfield J | Areteva R&D, Nottingham, UK; |
| Cunoosamy D | Sanofi, Cambridge, USA |
| D’Amico A | University of Rome ‘Tor Vergata’, Rome Italy; |
| Dahlén B | Department of Respiratory Medicine, Karolinska University Hospital & Centre for Allergy Research, Karolinska Institutet, Stockholm, Sweden |
| Dahlén S E | Institute of Environmental Medicine, Centre for Allergy Research, Karolinska Institutet, and Department of Respiratory Medicine, Karolinska University Hospital, Stockholm, Sweden |
| De Meulder B | European Institute for Systems Biology and Medicine, CNRS-ENS-UCBL-INSERM, Lyon, France; |
| Djukanovic R | NIHR Southampton Respiratory Biomedical Research Unit and Clinical and Experimental Sciences, Southampton, UK; |
| Erpenbeck V J | Translational Medicine, Respiratory Profiling, Novartis Institutes for Biomedical Research, Basel, Switzerland; |
| Erzen D | Boehringer Ingelheim Pharma GmbH & Co. KG; Biberach, Germany |
| Fichtner K | Boehringer Ingelheim Pharma GmbH & Co. KG; Biberach, Germany |
| Fleming L J | National Heart and Lung Institute, Imperial College, London, UK; Royal Brompton and Harefield NHS trust, UK |
| Formaggio E | *Previously CROMSOURCE, Verona Italy* |
| Fowler S J | Division of infection, immunity and respiratory medicine, School of biological sciences, University of Manchester, Manchester University NHS Foundation Trust, Manchester Academic Health Science Centre, Manchester, United Kingdom |
| Frey U | University Children’s Hospital, Basel, Switzerland; |
| Gahlemann M | Boehringer Ingelheim (Schweiz) GmbH,Basel, Switzerland; |
| Geiser T | Department of Respiratory Medicine, University Hospital Bern, Switzerland; |
| Goss V | NIHR Respiratory Biomedical Research Unit, University Hospital Southampton NHS Foundation Trust, Integrative Physiology and Critical Illness Group, Clinical and Experimental Sciences, Sir Henry Wellcome Laboratories, Faculty of Medicine, University of Southampton, Southampton, UK; |
| Guo Y | Data Science Institute, Imperial College, London, UK; |
| Hashimoto S | Academic Medical Centre, University of Amsterdam, Amsterdam, The Netherlands; |
| Haughney J | International Primary Care Respiratory Group, Aberdeen, Scotland; |
| Hedlin G | Dept. Women’s and Children’s Health & Centre for Allergy Research, Karolinska Institutet, Stockholm, Sweden; |
| Hekking P W | Academic Medical Centre, University of Amsterdam, Amsterdam, The Netherlands; |
| Higenbottam T | Allergy Therapeutics, West Sussex, UK; |
| Hohlfeld J M | Fraunhofer Institute for Toxicology and Experimental Medicine, Hannover, Germany |
| Holweg C | Respiratory and Allergy Diseases, Genentech, San Francisco, USA |
| Horváth I | Semmelweis University, Budapest, Hungary |
| Howarth P | NIHR Southampton Respiratory Biomedical Research Unit, Clinical and Experimental Sciences and Human Development and Health, Southampton, UK |
| James A J | Institute of Environmental Medicine, Centre for Allergy Research, Karolinska Institutet, Stockholm, Sweden; |
| Knowles RG | Knowles Consulting Ltd, Stevenage. UK; |
| Kolmert J | Institute of Environmental Medicine, Centre for Allergy Research, Karolinska Institutet, Stockholm, Sweden |
| Konradsen J | Dept. Women’s and Children’s Health & Centre for Allergy Research, Karolinska Institutet, Stockholm, Sweden |
| Krug N | Fraunhofer Institute for Toxicology and Experimental Medicine, Hannover, Germany; |
| Lazarinis N | Department of Respiratory Medicine, Karolinska University Hospital & Centre for Allergy Research, Karolinska Institutet, Stockholm, Sweden |
| Li C-X | Department of Medicine Solna, Karolinska Institutet, Stockholm, Sweden |
| Loza M J | Janssen R&D, LLC, Spring House, PA, USA |
| Lutter R | Academic Medical Centre, University of Amsterdam, Amsterdam, The Netherlands; |
| Manta A | Roche Diagnostics GmbH, Mannheim, Germany |
| Masefield S | European Lung Foundation, Sheffield, UK; |
| Maitland-van der Zee Anke-Hilse | Department of Respiratory Medicine, Amsterdam UMC, University of Amsterdam, Amsterdam, The Netherlands; |
| Matthews J G | Respiratory and Allergy Diseases, Genentech, San Francisco, USA; |
| Mazein A | European Institute for Systems Biology and Medicine, CNRS-ENS-UCBL-INSERM, Lyon, France |
| Middelveld R J M | Centre for Allergy Research, Karolinska Institutet, Stockholm, Sweden |
| Miralpeix M | Almirall, Barcelona, Spain; |
| Montuschi P | Università Cattolica del Sacro Cuore, Milan, Italy; |
| Murray C S | Division of infection, immunity and respiratory medicine, School of biological sciences, University of Manchester, Manchester University NHS Foundation Trust, and Manchester Academic Health Science Centre, Manchester, United Kingdom |
| Musial J | Dept. of Medicine, Jagiellonian University Medical College, Krakow, Poland |
| Mumby, S | National Heart and Lung Institute, Imperial College, London, UK |
| Myles D | Respiratory Therapeutic Unit, GSK, London, UK; |
| Nordlund B | Dept. Women’s and Children’s Health & Centre for Allergy Research, Karolinska Institutet, Stockholm, Sweden |
| Pandis I | Data Science Institute, Imperial College, London, UK |
| Pavlidis S | National Heart and Lung Institute, Imperial College, London, UK |
| Postle A | University of Southampton, UK |
| Powel P | European Lung Foundation, Sheffield, UK; |
| Praticò G | CROMSOURCE, Verona, Italy |
| Puig Valls M | CROMSOURCE, Barcelona, Spain |
| Rao N | Janssen R&D, LLC, Spring House, PA, USA |
| Reinke S | Institute of Environmental Medicine, Karolinska Institutet, Stockoholm, Sweden |
| Riley J | Respiratory Therapeutic Unit, GSK, London, UK; |
| Roberts A | Asthma UK, London, UK; |
| Roberts G | NIHR Southampton Respiratory Biomedical Research Unit, Clinical and Experimental Sciences and Human Development and Health, Southampton, UK; |
| Rowe A | Janssen R&D, UK; |
| Sandström T | Dept of Public Health and Clinical Medicine, Umeå University, Umeå, Sweden; |
| Schofield JPR | Centre for Proteomic Research, Institute for Life Sciences, University of Southampton, Southampton, UK |
| Seibold W | Boehringer Ingelheim Pharma GmbH, Biberach, Germany |
| Shaw D E | Respiratory Research Unit, University of Nottingham, UK; |
| Sigmund R | Boehringer Ingelheim Pharma GmbH & Co. KG; Biberach, Germany |
| Singer F | Pediatric Respiratory Medicine, Department of Pediatrics, Inselspital, Bern University Hospital, University of Bern, Bern, Switzerland. |
| Skipp P J | Centre for Proteomic Research, Institute for Life Sciences, University of Southampton, Southampton, UK |
| Smicker M | Sanofi, Cambridge, USA |
| Sousa A R | Respiratory Therapeutic Unit, GSK, London, UK; |
| Sparreman-Mikus M | Department of Medicine Huddinge, Karolinska Institutet, and Department of Respiratory Medicine, Karolinska University Hospital, Stockholm, Sweden |
| Sterk P J | Academic Medical Centre, University of Amsterdam, Amsterdam, The Netherlands; |
| Ström M | Department of Medicine Solna, Karolinska Institutet, Stockholm, Sweden |
| Sun K | Data Science Institute, Imperial College, London, UK |
| Thornton B | MSD, USA |
| Uddin M | AstraZeneca BioPharmaceuticals R&D, Gothenburg, Sweden |
| Versi A | National Heart and Lung Institute, Imperial College, London, UK |
| Vestbo J | Centre for Respiratory Medicine and Allergy, Institute of Inflammation and Repair, University of Manchester and University Hospital of South Manchester, Manchester Academic Health Sciences Centre, Manchester, United Kingdom |
| Vissing N H | COPSAC, Copenhagen Prospective Studies on Asthma in Childhood, Herlev and Gentofte Hospital,  University of Copenhagen, Copenhagen, Denmark; |
| Wagers S S | BioSci Consulting, Maasmechelen, Belgium |
| Wheelock A | Respiratory Medicine Unit, Department of Medicine Solna and Center for Molecular Medicine, Karolinska Institutet, Stockholm, Sweden; and Department of Respiratory Medicine and Allergy, Karolinska University Hospital Solna, Stockholm, Sweden; |
| Wheelock C E | Institute of Environmental Medicine, Centre for Allergy Research, Karolinska Institutet, Stockholm, Sweden; |
| Wilson S J | Histochemistry Research Unit, Faculty of Medicine, University of Southampton, Southampton, UK; |
| Yasinska V | Department of Medicine Huddinge, Karolinska Institutet, and Department of Respiratory Medicine, Karolinska University Hospital, Stockholm, Sweden |
| Zounemat Kermani, N | Data Science Institute, Imperial College, London, UK |
